# Multi-dimensional safety risk assessment on coal mines under the profitability dilemma

**DOI:** 10.1038/s41598-023-29795-3

**Published:** 2023-02-15

**Authors:** Qi You, Qingguo Yao, Ruixin Song, Kai Yu, Cuicui Xu, Haiying Cao

**Affiliations:** 1 Department of Safety Engineering, College of Safety and Environmental Engineering, Shandong University of Science and Technology, 579 Qianwangang Road, Huangdao District, Qingdao, 266590 Shandong Province People’s Republic of China; 2 Department of Emergency Management of Jiangxi Province, Nanchang, 330001 China; 3 State Key Laboratory of Mining Disaster Prevention and Control Co-Founded By Shandong Province and the Ministry of Science and Technology, Shandong University of Science and Technology, Qingdao, 266590 China; 4 Jiangxi Tungsten Holding Group Company Limitend, Nanchang, 330038 China; 5 State Key Laboratory Cultivation Base for Gas Geology and Gas Control (Henan Polytechnic University), Henan Polytechnic University, Jiaozuo, 454000 China

**Keywords:** Natural hazards, Engineering

## Abstract

China is a major coal producer, with huge differences in coal production and safety situations between the South and the North. Taking province A as an example, its coal enterprises have low output, poor efficiency, backward equipment, and low-quality personnel. The output accounts for 0.08% of the country, and the number of deaths accounts for 2.2% of the country, the safety situation of coal enterprises in province A is severe. In order to study the safety risk situation of coal mines under difficult conditions, this paper screens 98 factor indexes including multiple subjects such as enterprise managers, front-line workers, government supervisors, external environment, work quality, stress factors, economic factors, and other dimensions. For different data, the indicator weights were calculated using triangular fuzzy number, entropy weight method, CRITIC method, and three rough set methods in a total of six methods. The comprehensive weights of the indicators were obtained using the proposed new comprehensive weight method. The current situation of safety work of four coal mining enterprises and three levels of government supervision departments was evaluated, and the evaluation results were compared with other existing data to verify the reliability of the evaluation system. The horizontal comparison of the evaluation results indicates the main problems of each subject; the longitudinal comparison points out the problems that need to be solved with the assistance of higher-level enterprises and the central government, and corresponding suggestions for coal mining enterprises and government departments are put forward to reduce the safety risks of troubled coal mining enterprises.

## Introduction

China is a major coal producer, and the coal production reached up to 3.84 billion tons in 2020, accounting for 51% of the world’s total (BP 2021). In addition, China's coal consumption has always ranked first in the world, and the ratio of coal to primary energy has always been higher than 50%. With the development of coal production, there have occurred many fatal accidents in China’s coal industry. Since 1949, there have been 24 accidents with more than 100 casualties for each occurring in the coal industry, totally causing 3780 deaths. In order to reduce coal production safety accidents, China has closed many coal mines with low production technology and achieved great progress in work safety. However, due to the lack of natural gas and oil, coal is still the main energy in China. Due to the external environment, such as the war between Russia and Ukraine, China's energy sources are under constant strain. The government has repeatedly urged coal mining enterprises to increase production capacity and ensure energy supply. The current situation that China's coal production and coal consumption are at historically high levels will not be fundamentally changed. It is still an important task for China to improve the safety management of coal mining enterprises and avoid coal mine safety accidents.

According to data from the National Mine Safety Supervision Bureau, there are 4,495 coal mines in China in 2020, 4,173 of which are underground mines, and the remaining 358 are open-pit coal mines, all of which are located in northern China. With the concerted efforts of many parties, the coal production safety situation in China continues to improve. The degree of mechanization of large coal mines in China has increased from 32.5% in 1978 to 97.9% in 2020, and the per capita coal production has reached 1000t/a, 6.3 times and 2.6 times higher than 137 t/a in 1978 and 276 t/a in 1999, respectively. In 2019, there were 170 coal mine accidents in China and 316 people died. Compared with the peak of 7016 people in 1994, the death toll dropped by 95.5%^1^. The national coal mine mortality rate per million tons continues to decrease, and is 0.044 in 2021, a drop of 98.82% from 2005. However, the death rate per million tons in Southern province A has been higher than 1. Due to the wide variation of coal seam occurrence conditions in different regions of China, the unbalanced production level of coal mines in various regions is prominent. There are super-large mines with an annual output of over 10 million tons in the north, and micro-coal mines with an annual output of 30,000 tons in the south. In 2021, China has a total of 22 coal-producing provinces, and the coal production of the four northern provinces accounts for 79.9% of the country's total. At present, China's various technical standards are dominated by northern coal mining enterprises. A meeting material of the National Mine Safety Supervision Bureau shows that as of January 2021, all mining areas in northern China have used fully mechanized mining, started the construction of 71 intelligent demonstration mines, built 183 intelligent mining faces, and are exploring the construction of unmanned mining working face. Benefiting from the upgrading of equipment, the labor intensity of underground front-line workers is much lower. The front-line workers basically have a college degree or even a bachelor's degree, and the quality of personnel and industry wages have been greatly improved. After the Shaanxi Coal Industry Group in northern China carried out the intelligent transformation of coal mines, its coal output increased by 70 million tons, the number of excavation faces decreased by 42%, and the cumulative number of underground workers was reduced by 14,000. So far, the national intelligent coal mines have eliminated major accidents, and the death rate per million tons is 0.024, which is 50% lower than the national average. In Heilongjiang province in northern China, for example, the intelligent mining production capacity accounts for about one-third of the country's total output, and the death rate per million tons is only one-sixth of the national average level of coal mines. In contrast, the southern region, represented by province A, cannot effectively adopt mechanized and informationized coal mining equipment due to the thickness of coal seams, dense faults, and other reasons. As a result, the gap between them and the national standard is widening, and the pressure to be regulated by government departments and fined is high. With limited production capacity, some of the required technical changes have increased the pressure on the profitability of enterprises. The labor intensity of enterprise personnel in the south is high, and the wages are low. Only farmers with low education levels, difficult life, and older age are willing to work in the mine. There are huge differences between the south and the north in terms of technical equipment, mining scale, safety investment, personnel quality, and safety production level. In 2020, the death rate per million tons was 27.4 times that of the country and 164.4 times that of Heilongjiang Province. Zhou^2^ divided China into five regions based on the million-ton mortality data of each province in China from 2001 to 2019. Among them, the southern region, where province A is located, has the lowest safety level. A quantitative study of the degree of spatial variation in production levels and the driving factors indicated that the mining environment has the greatest influence on the safety level of coal mines in the southern region, and that regulatory indicators, law enforcement, and economic environment have a strong nonlinear effect on improving the safety level of coal mining enterprises. This result confirms the production safety dilemma faced by coal mining enterprises in province A from another perspective.

In Webbers and Oxford Dictionary, risk refers to the possibility of facing danger or suffering loss, and generally refers to the possibility of production accidents in coal mining enterprises. The production conditions of underground coal mines are complex, the conditions change greatly, and there is a high degree of uncertainty^3^. In order to study the indicators of influencing factors of coal mine safety risk, some experts, based on the analysis of coal mine accidents, believe that the influencing factors of coal mine safety risk mainly include 7 aspects, such as equipment factors, safety technology factors, natural factors, safety management factors, legal supervision factors, economic factors, and employee factors. In addition, there are a lot of interactions among the 7 categories of factors^4,5^. In addition, some scholars have analyzed the influence of factors such as the level of enterprise informatization^6^, employee safety management satisfaction^7^, coal mining technology^8^, and coal mine safety supervision^9^ on enterprise safety risks. Ma et al.^10,11^ took government supervision as the starting point to study the relationship between government behavior and enterprise safety accidents.

In the comprehensive evaluation of safety risks, determining the weight of indicators is an important part of building an evaluation system. In the current field of coal mine safety evaluation in China, AHP is the mainstream method for calculating weights^12,13^, and it has been used by many scholars to calculate indicator weights^14,15^. However, the AHP method has certain limitations, and it is difficult for experts to make accurate judgments about the relative importance of indicators. For this reason, Zhang^16^, He^17^, and Qi^18^ improved the AHP by combining fuzzy evaluation methods such as the triangular fuzzy number and GSPA. In addition, TOPSIS^19^, information entropy and unconfirmed measure (UM)^20^, Gaussian affiliation function^21^, and triangular fuzzy number method^18,22^ have also been used to calculate the weights of index factors in the evaluation system. All these methods have their own advantages and disadvantages. In order to maximize the advantages of the methods and reduce the influence of method disadvantages, Tian^23^ and Chen^24^ combined rough set theory with multilevel fuzzy judgment method and Matter-Element analysis, respectively, and JISKANI^12^ used entropy weight method and gray clustering method, Qiao Wanguan^25^ used DEA- BBC model and DEA-Malmquist index as weight calculation methods to determine the weight size of the indicators and then analyze and evaluate the research object.

For the safety risk management and control of coal mines, Jiang^26^ used a large amount of data to build a coal mine risk data prediction model by optimizing the BP neural network. M. Ilyashov^27^ proposed a systematic management method of coal mine risk based on the safety risk assessment of each working link, and focused on the work pressure of managers in coal mine enterprises^18^. Jiskani^28–30^ took the Basquitaine mines as an example to study the influence of government policies, safety supervision, miners' safety status, and professional quality on the sustainable development of mines. Han^31^ studied the influence of factors such as personal characteristics and the social environment of mine employees on the effect of enterprise safety commitment, which provided a new perspective for improving the efficiency of enterprise safety management. Studies have shown that good safety management^32,33^ and targeted investment in manpower and material resources^34^ can effectively reduce corporate safety risks. Among the many influencing factors, some factors are difficult to effectively control due to the external environment, and have a greater impact on enterprise safety risks than other factors. In the field of safety risk management, some academics assess various safety risks of enterprises were evaluated to provide supporting suggestions for enterprise safety production work and government safety supervision work^35,36^.

## Overview of the coal mine production safety situation in Province A

Province A is located in the southeast of China, and the geological conditions of coal seams are complex. Five major disasters have occurred in this area. In the past, there have been safety accidents in which more than 100 people died. As the central government gradually phases out outdated production capacity with low production capacity, the number of coal mines in province A has dropped from 885 in 2005 to 31 in 2020. In 2019, province A produced 5.0361 million tons of coals. Compared with the consumption of 79.9594 million tons that year, the coal self-sufficiency rate was only 6.3%. The existing 7 state-owned coal mines (belonging to the same group company) have an average production capacity of 387,000 tons/year (only 1 has a mechanized coal mining face, no mechanized driving face), and 24 private coal mines have a production capacity of 86,700 tons/year (both are blasting coal mining and excavation). This is very different from the national average level of 1.182 million tons per year (Table 1). In 2020, the output of coal mines in province A accounted for 0.08% of the country, and the death toll accounted for 2.2% of the country. The provincial government of province A is under great pressure in the production safety assessment in the past years, and has the will to withdraw from the coal mining industry as a whole, and has decided to withdraw from all private coal mines by 2025. However, state-owned coal mining enterprises also need to consider the value of the assets of the state-owned enterprises (the provincial group is a listed company with total assets of 8.387 billion RMB), the employment of cadres and workers (a coal mine with an annual production capacity of less than 900,000 tons in province A supports more than 3,000 employees), the pressure of employee resettlement after the withdrawal of coal mines (the cost of employee resettlement for a provincial coal mine to be withdrawn from province A in 2020 is more than 200 million RMB), and other responsibilities such as energy supply. Therefore, although the seven state-owned coal mining enterprises are willing to withdraw, they can only withdraw in an orderly and reasonable manner without an uncertain timetable according to the needs of energy supply guarantee and the safety production situation of the industry.

**Table 1:** Comparison of the safety production situation of coal mines between China and Province A (2020).

	Total quantity of coal mines (including idle coal mines)	Production capacity (ten thousand tons per year)	Single mine production capacity (ten thousand tons per year)	Ratio of standardized coal mines above grade II	Mortality of coal mine accidents in 2020	Mortality rate per million tons in 2020
China	4495	541,208	118.2	75.8%	225	0.058
Province A	31	479	15.5	9.7%	5	1.59

**Figure 1:**
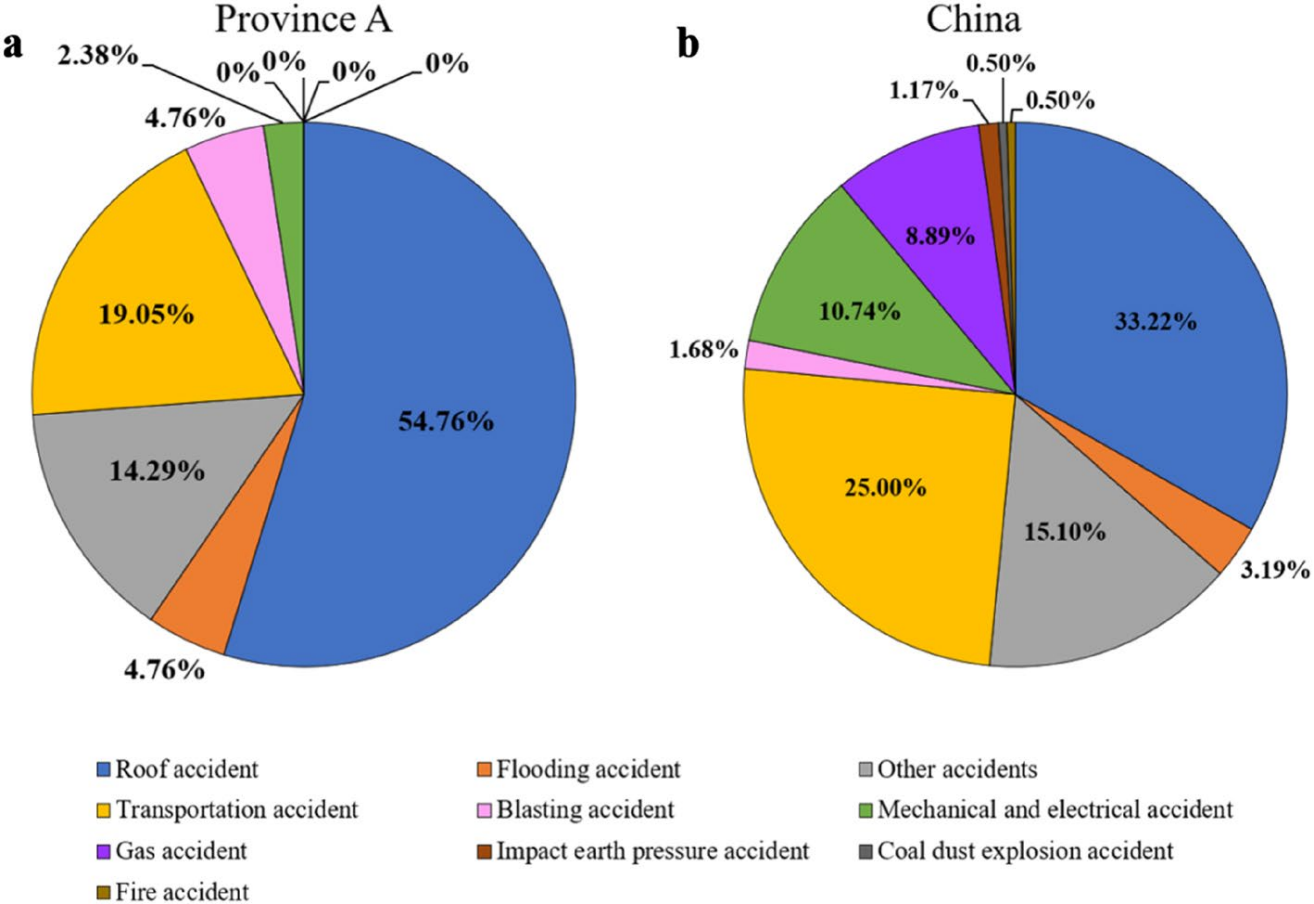
Comparison of the number of types of accidents in coal mines between China and Province A (2016–2020).

**Figure 2:**
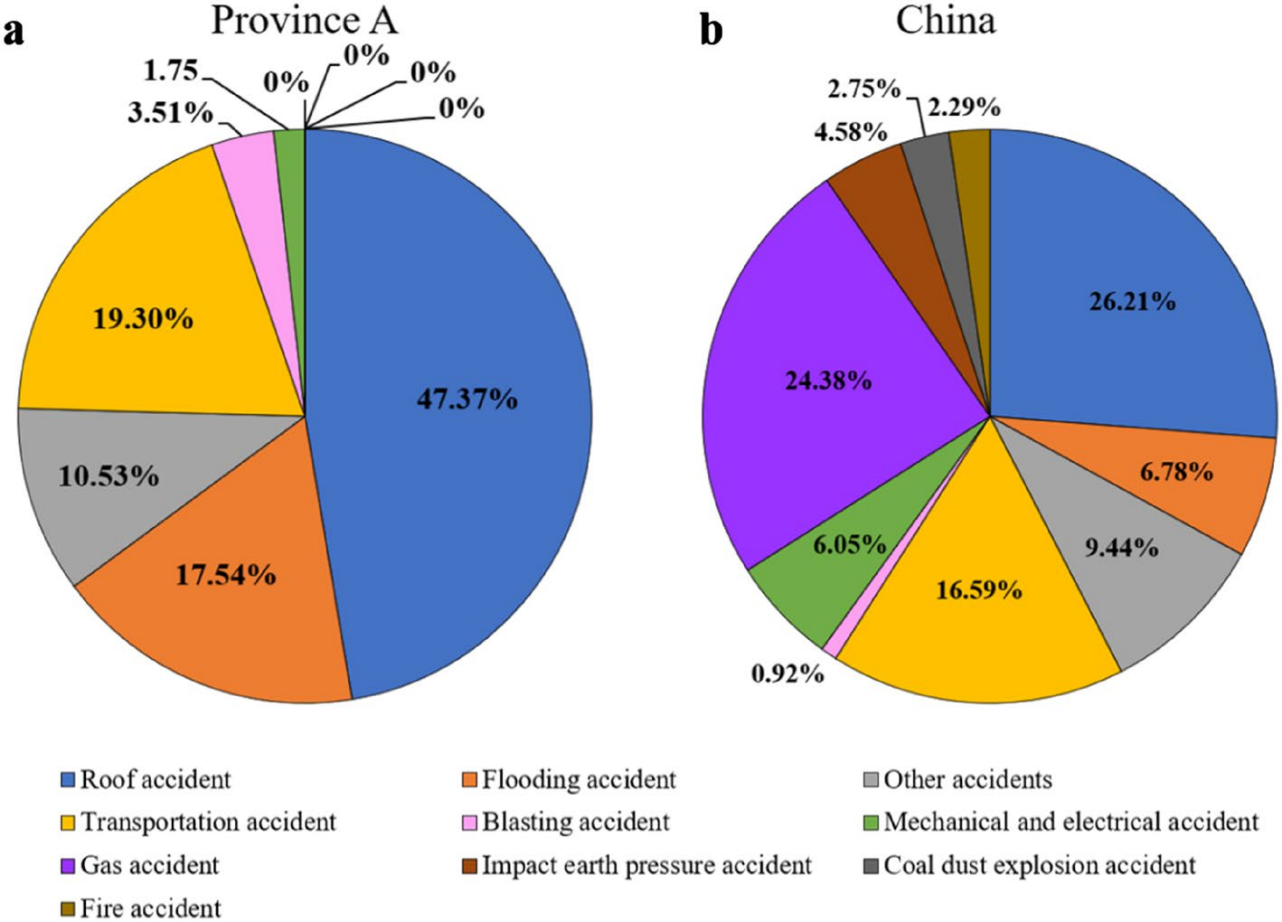
Comparison of the Mortality by type of accident in coal mines between China and Province A (2016–2020).

From 2016 to 2021, there were 42 accidents and 57 deaths in province A, and 596 accidents and 1091 deaths in China. Comparing the types of accidents and fatalities in province A with the national data, it is found that the death ratios of three types of accidents, i.e., roof accidents, flooding accidents, and blasting accidents, were 1.8 times, 2.6 times and 3.8 times the national average, respectively, and the number of accidents in three types was 1.6 times, 1.5 times and 2.8 times of the national average (Figs. 1 and 2). In the investigation report of 42 accidents in province A, the causes of all accidents were related to personnel's illegal operations. In a province-wide survey in 2021, there were 10,945 coal mine employees in province A, of which 62.3% had junior high school education, 30.2% had high school education, 7.5% had a college education or above, and the average age was 53.5 years old. In the third quarter of 2022, the listed parent companies of the seven state-owned coal mines in province A have lost 225 million RMB. When the coal price is high, the company is still unable to achieve profitability. In daily work, the author conducted in-depth exchanges with government regulators at all levels and enterprise managers in the coal mine industry in province A. It was found that all parties recognized that the coal mines in province A had a low level of informatization and mechanization, the aging of employees was serious, and all types of illegal and illegal activities have not been effectively eliminated. For a long time to come, the status quo of state-owned coal mining enterprises in province A will be difficult to reverse, and the situation of coal mining enterprises in many other southern provinces in China is similar to that of province A. If only considering a single dimension such as work implementation quality while ignoring the impact of external “soft environment” such as economic factors, policy factors, ore prices, local financial resources, and supervision on enterprise safety risks, the established evaluation index system will be difficult to reflect the actual situation of enterprises, which is not broadly representative and is difficult for large-scale promotion and application. We conducted a targeted study on the safety risk management and control of the state-owned coal mines in province A with a difficult situation, so as to reduce the number of accidents and deaths in coal mines, which is of practical and important significance to the safety production of coal mines in province A and even southern China.

## Construction of a multi-dimensional evaluation system for safety risk

### Construction of index system

Safety risks are closely related to many things. These things (indicators) will affect each other and have a very complicated relationship with safety risks. However, everyone's cognition and understanding of things are quite different. Therefore, the safety risk is a subjective evaluation judgment on an objective basis. Based on many forums with the safety regulation cadres of coal mines at provincial, municipal, and county levels, the experts employed by the supervision departments, the administrative staff of state-owned key mines in province A, and the front-line workers, and by referring to a wide range of literature and data, the authors believe that seven major categories of subjects, including enterprise managers, front-line workers, government regulators, intermediary evaluation agencies, equipment manufacturers, scientific research scholars, and the external social environment, have a huge impact on the safety risks of mining enterprises. Among them, intermediary evaluation agencies, equipment manufacturers, scientific research scholars, and other subjects affect the safety work of enterprises through the role of enterprise managers, front-line workers, and government supervisors.

**Figure 3:**
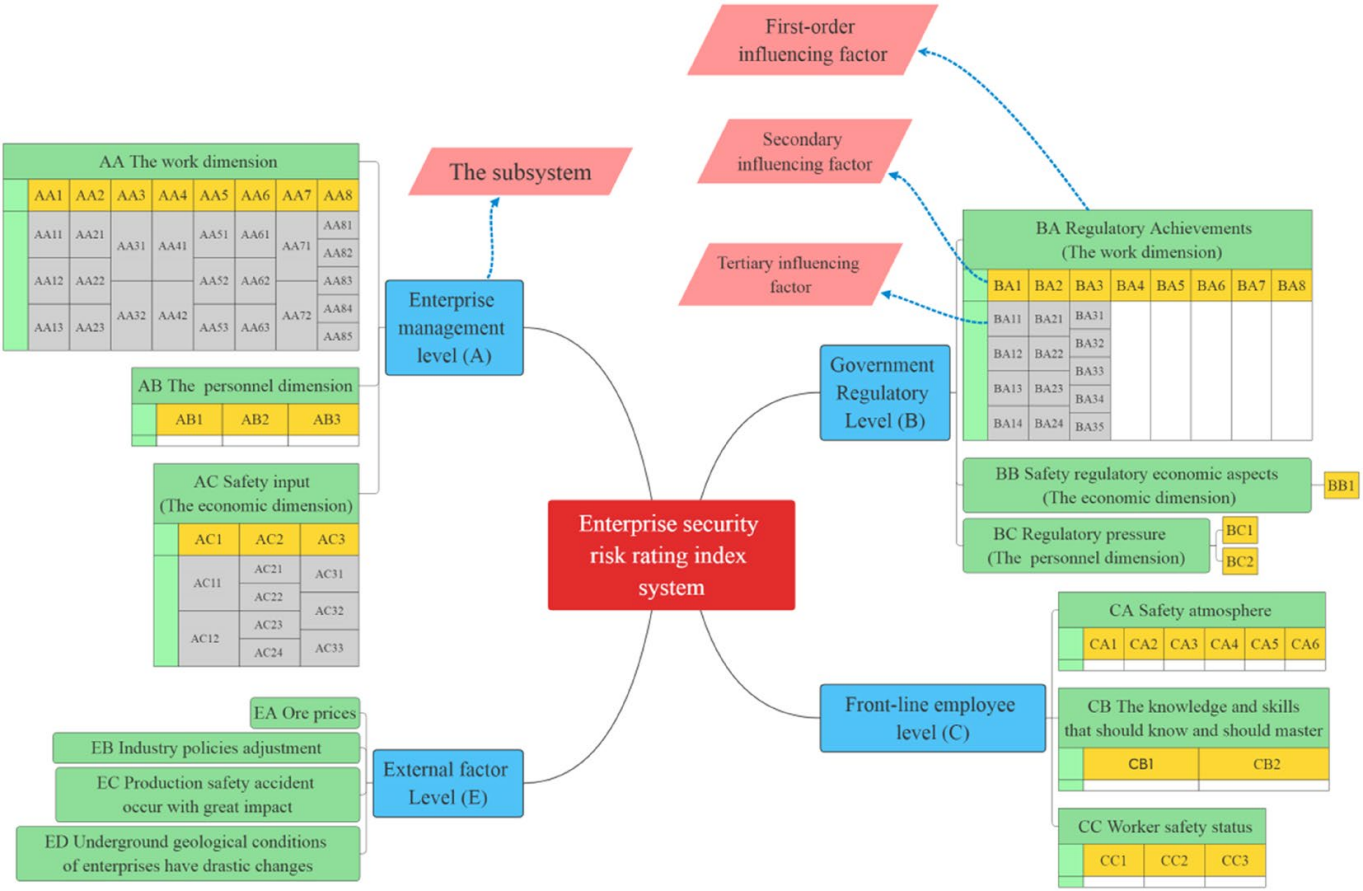
Safety risk evaluation index system of enterprises.

In order to more scientifically and accurately perceive enterprise safety risks, in addition to considering the "geological conditions", "equipment and technology level", "safety management capability", "the personal quality of employees", "investment in safety production" and other "hardware level" of coal mines, it is also necessary to take into account the individual income demands of various groups from a "people-oriented" perspective (personal economic level) and the need for government regulators to "exempt those that have diligently fulfilled their duties from liabilities" (pressure level) to consider the work objectives of state-owned enterprises and government managers to "protect people's livelihood", "maintain stability", "maintain employment" and "maintain operation" from a social and political responsibility perspective. We should start from the entire safety production system of mining enterprises, analyze the "principal contradictions" that need to be overcome in safety risk management and control, perceive changes in various risks in a timely manner, and formulate measures to control risks and kill accidents in the cradle. Relying on the "Standardization of Coal Mine Safety Production Management System", "Province A Coal Industry Group 2022 Safety Work Assessment Measures" and the research results of existing scholars^4–10,12^, this study followed the principle of “multi-subject and multi-dimension” to determine the four subsystems related to the safety risk of enterprises, namely, government, enterprise, employee, and external factors. For the enterprise administrator and the government supervisors, multiple evaluation indexes in terms of work, pressure, and economy were determined. From the dimensions of safety climate, safety skills, and safety state, we analyzed the safety risk of enterprise employees and gave special consideration to the effects of some external factors including industry policies and coal price on the safety risk of enterprises, thereby establishing the evaluation index system. This system consists of 4 subsystems, 13 first-level indexes, 36 s-level indexes, and 45 third-level indexes. The index system structure is shown in Fig. 3, and the specific indicators are shown in Table 2 (A, AA, AA1, and AA11 are the numbers of the first, second, and third-level indicators of the subsystem, respectively).

**Table 2:** List of indicators for enterprise safety risk assessment.

First-level influencing factor	Secondary influencing factor	Tertiary influencing factor
AA Safety management quality	AA1 Risk grading control work quality	AA11 Safety risk identification and assessment
		AA12 Safety risk control measures
		AA13 Risk control safeguards
	AA2 Hidden trouble investigation and management quality	AA21 Hidden trouble investigation work quality
		AA22 Quality of hidden trouble governance
		AA23 Hidden trouble rectification supervision and management quality
	AA3 General quality of regulations	AA31 Perfection of enterprise rules and regulations
		AA32 Operability of enterprise rules and regulations
	AA4 Managerial personnel management ability	AA41 Quality of management personnel
		AA42 Managerial leadership
	AA5 Strength of rewards and punishments for workers	AA51 System implementation
		AA52 Level of punishment
		AA53 Incentive level
	AA6 Quality of safety training	AA61 Training of teachers
		AA62 Training methods attractive
		AA63 Training assessment strength
	AA7 Quality of site management	AA71 Technical solution quality
		AA72 Site construction quality
	AA8 Feedback summary change strength	AA81 Finds common problems in time
		AA82 Finds major problems in time
		AA83 Formulate effective plans in a timely manner
		AA84 Timely implementation of the program
		AA85 Feedback improvement in time
AB Management personnel operating pressure	AB1 Management personnel corporate earnings pressure	
	AB2 Management personnel enterprise stability pressure	
	AB3 Manage personnel safety pressure	
AC safety input	AC1 Daily safety engineering input	AC11 New technologies and equipment
		AC12 Safety training input
	AC2 Enterprise output value	AC21 Unsaturation rate of operating personnel
		AC22 Enterprise operating site unsaturated rate
		AC23 Enterprises to conceal the location of the impulse
		AC24 Enterprise overmanned well impulse
	AC3 Enterprise surplus	AC31 Employee salary
		AC32 Superior financial assistance
		AC33 Superior financial pressure
BA Regulatory achievements	BA1 Frequency of supervision	BA11 Frequency of national supervision
		BA12 Provincial regulatory frequency
		BA13 Municipal regulatory frequency
		BA14 County level supervision frequency
	BA2 Degree of punishment	BA21 Administrative penalty
		BA22 Ordered to stop production for rectification
		BA23 Letter for appointment
		BA24 Penalties for major hidden dangers
	BA3 Supervision capability	BA31 Expert participation
		BA32 Supervises the quality of cadres
		BA33 Supervision cadre professional skills
		BA34 Supervision business training
		BA35 Ability to detect significant hazards
	BA4 Supervision initiative	
	BA5 Modern regulatory tools	
	BA6 Government supervision check hidden dangers	
	BA7 Government supervision finds problems	
	BA8 Conducts safety awareness	
BB Safety regulatory economic aspects	BB1 Sector operating economic pressure	
BC Regulatory pressure	BC1 Social stability pressure	
	BC2 Accident accountability pressure	
CA Safety atmosphere	CA1 Stop unsafe behavior of workmates	
	CA2 Report safety risks of other posts	
	CA3 Reveres all kinds of safety regulations	
	CA4 Income satisfaction	
	CA5 Satisfaction degree of working environment	
	CA6 Work intensity satisfaction	
CB The knowledge and skills that should know and should grasp	CB1 Detect safety risks	
	CB2 Self-management ability	
CC Worker safety status	CC1 Safety concept	
	CC2 Worker’s safety awareness	
	CC3 Worker’s "three violations"	

### Weight calculation method

Weighting methods can be divided into empirical weighting and mathematical weighting. Empirical weighting, also known as qualitative weighting, is the direct estimation of the weight by experts. According to the source form of the original data, mathematical weighting can be divided into subjective weighting method and objective weighting method. The original data of the subjective weighting method comes from experts’ judgment based on experience; while the raw data of the objective weighting method is formed without seeking expert opinion. In general, subjective weights rely on the experience of experts to judge the importance of factor indexes, but are limited by the cognitive level and work experience of experts, so it is inherently limited; objective weighting is derived from objective data, but it will change with the variation of evaluation objects, so it is less stable than subjective weighting, which causes some calculation results to be far from the actual situation.

Each mathematical weighting method has its own focus and advantages and disadvantages, and it is difficult to compare which one is better. In order to take into account the advantages of subjective and objective weights, various factors are considered. For subsystems and primary indicators, they involve multiple aspects and dimensions, and it is difficult to collect relevant data. Only a few experts have the importance to determine relevant indicators. Thus, the importance is scored by experts, and the weights of subsystems and primary indicators are calculated by the triangular fuzzy number method. For the secondary and tertiary indicators, the current status of each work is investigated by issuing a questionnaire (the questionnaire is a Likert scale question, and the questionnaire questions correspond to the indicators one by one). The questionnaire data were analyzed using the entropy weighting method, CRITIC method, and five objective weighting methods such as Risk, Pos, and Neg of the rough set. Five weights of the secondary and tertiary indicators were derived. Then the comprehensive weight calculation method based on Euclidean distance information entropy and the rough set was used to calculate the comprehensive weights of secondary indicators and tertiary indicators.

#### Subjective weighting method—triangular fuzzy number

Some scholars have investigated the triangular fuzzy number theory in venture investment and equipment manufacturing domains. In this paper, the factor indexes (subsystems, first-level indicators) that need to be calculated were used as the factor index set X = {X_1_, X_2_, X_3_,…X_m_}, in which m denotes the number of factor indexes. Invite several experts to grade three scores $(l,g,h)$, for each index according to the importance degree, which represents the lower limit value, the most possible value, and the upper limit value of the importance degree of the index, respectively. Assuming that denotes the score of the j-th factor index by the i-th expert using the triangular fuzzy number method as ${\tilde{\text{q}}}_{{{\text{ij}}}} = (l_{ij} ,g_{ij} ,h_{ij} )$, ${\tilde{x}}_{j}$ denotes the fuzzy comprehensive score of the jth factor index, and n denotes the number of experts. Based on previous research results (Qi 2014), the weights of the subsystems and the first-level indexes can be calculated with the triangular fuzzy number method. The details are as follows:

1 $$\tilde{x}_{{\text{j}}} = \left[ {\sum\limits_{i = 1}^{n} {l_{ij} \sum\limits_{i = 1}^{n} {g_{ij} } \sum\limits_{i = 1}^{n} {h_{ij} } } } \right] + \left[ {\sum\limits_{i = 1}^{n} {\sum\limits_{j = 1}^{m} {l_{ij} ,\sum\limits_{i = 1}^{n} {\sum\limits_{j = 1}^{m} {g_{ij} } } ,\sum\limits_{i = 1}^{n} {\sum\limits_{j = 1}^{m} {h_{ij} } } } } } \right]^{ - 1} \approx \left[ {\frac{{\sum\limits_{j = 1}^{m} {l_{ij} } }}{{\sum\limits_{i = 1}^{n} {\sum\limits_{j = 1}^{m} {h_{ij} } } }},\frac{{\sum\limits_{j = 1}^{m} {g_{ij} } }}{{\sum\limits_{i = 1}^{n} {\sum\limits_{j = 1}^{m} {g_{ij} } } }},\frac{{\sum\limits_{j = 1}^{m} {h_{ij} } }}{{\sum\limits_{i = 1}^{n} {\sum\limits_{j = 1}^{m} {l_{ij} } } }}} \right]$$

The left and right expected values of ${\tilde{h}}_{j}$ can be expressed as:

2 $$I_{L} ({\tilde{x}}_{j} ) = \frac{{l_{j} + m_{j} }}{2},I_{R} ({\tilde{x}}_{j} ) = \frac{{m_{j} + h_{j} }}{2}$$

φ was taken as the pessimistic-optimistic coefficient and φ = 0.5 was set as the median number. Accordingly, φ > 0.5 indicates the pessimistic tendency of the decision-maker, and φ < 0.5 indicates the optimistic tendency of the decision-maker. In this study, φ = 0.5.

3 $$I(\tilde{x}_{j} ) = \phi \times I_{L} (\tilde{x}_{j} ) + 1 - \phi \times I_{{\text{R}}} (\tilde{x}_{j} ) = \frac{{l_{j} + 2m_{j} + h_{j} }}{4},\quad i \in I$$

Assuming the weight of the j-th index as ω_j_, in which $\sum\limits_{{{\text{j}} = 1}}^{{\text{m}}} {\omega_{{\text{j}}} } = 1$.

4 $$\omega_{{\text{j}}} = \frac{{I\left( {\tilde{x}_{j} } \right)}}{{\sum\limits_{j = 1}^{m} {I\left( {\tilde{x}_{j} } \right)} }},\quad i \in I$$

#### Objective weighting method—entropy weight method, CRITIC method, three rough set methods

In this paper, the entropy weight method, CRITIC method, and rough set (i.e., risk, Pos, and Neg) are used to calculate the weights of the secondary and tertiary indicators for the objective data obtained from the questionnaire survey. The entropy value is a measure of uncertainty. The greater the amount of information, the smaller the uncertainty and the smaller the entropy; the smaller the amount of information, the greater the uncertainty and the greater the entropy. Combined with the variation degree of each index, the weight calculation is carried out by using the information carried by the entropy value. The CRITIC method calculates the index weight according to the contrast strength and conflict of different data of the same index. The contrast strength is represented by the standard deviation. If the standard deviation of the data is larger, the fluctuation is greater, and the weight will be higher; the conflict is represented by the correlation coefficient. If the correlation coefficient value between the indicators is larger, the conflict is smaller, and its weight is also lower. When calculating the weight, the contrast intensity is multiplied by the conflicting index and normalized to obtain the final weight. The entropy weight method^37^ and CRITIC method^38^ have been studied in depth by many scholars, so this article will not repeat them. This article focuses on the three methods of Risk, Pos, and Neg in rough sets.

Rough set was proposed by Z. Pawlak in 1982, a professor of mathematics at Warsaw University of Technology in Poland. It is used to study incomplete data. It can effectively analyze and process incomplete and inaccurate data information, and mine hidden information from it to reveal the inner connection law. Rough set is a tool for dealing with fuzzy and uncertain problems, and has been widely used in knowledge discovery, machine learning, pattern recognition, data mining, expert system, decision analysis, and decision support. Suppose the quaternion S = (U, A, V, f) is a knowledge expression system, where U: U ≠ ∅ is a finite set of objects we are interested in, called the universe of discourse, which in this paper represents the information contained in the questionnaire (sample).

Definition: Given a knowledge base K = (U,Z), for each subset X ⊆ U and a hierarchic relation Z ∈ ind(K), we can define two subsets:

5 $$\begin{gathered} \underline {Z} X = \bigcup {\left\{ {Y \in U/} \right.} Z|Y \subseteq X\} ,\quad \hfill \\ \overline{Z}X = \bigcup {\left\{ {Y \in U/} \right.} Z|Y\bigcap X = \left. \phi \right\} \hfill \\ \end{gathered}$$

which are the lower approximate set of *X* and the upper approximate set of *Z*, respectively.

The set $bn_{Z} \left( X \right) = \overline{Z} X - \underline{Z} X$ is referred to as the border domain of Z, while $pos_{Z} \left( X \right) = \underline{Z} X$ and $neg_{Z} \left( X \right) = U - \overline{Z} X$ are referred to as the positive domain and negative domain of Z, respectively. Generally, Z can be neglected. $bn_{Z} \left( X \right)$, $pos_{Z} \left( X \right)$, and $neg_{Z} \left( X \right)$ can be denoted as $bn\left( X \right)$, $pos\left( X \right)$, and $neg\left( X \right)$, respectively.

In the single-parameter decision rough set, the global decision-making risk can be expressed as:

6 $${\mathcal{R}}_{\mathrm{B}}={\sum }_{\mathrm{x}\in {\mathrm{POS}}^{\mathrm{s}}}\left(1-\mathrm{P}(\mathrm{X}|[\mathrm{x}{]}_{\mathrm{B}})\right)\cdot {\uplambda }_{\mathrm{PN}}+{\sum }_{\mathrm{x}\in {\mathrm{BND}}^{\mathrm{S}}}\left(\mathrm{P}(\mathrm{X}|[\mathrm{x}{]}_{\mathrm{B}})\cdot {\uplambda }_{\mathrm{BP}}+(1-\mathrm{P}(\mathrm{X}|[\mathrm{x}{]}_{\mathrm{B}}))\cdot {\uplambda }_{\mathrm{BN}}\right)+{\sum }_{\mathrm{x}\in {\mathrm{NEG}}^{\mathrm{S}}}\mathrm{P}(\mathrm{X}|[\mathrm{x}{]}_{\mathrm{B}})\cdot {\uplambda }_{\mathrm{NP}}$$

where $\mathrm{B}\subseteq \mathrm{C}$. ${\mathcal{R}}_{\mathrm{B}}$ can be referred to as the global decision-making risk since it fully considers all possible risks. The loss function of the above equation is defined as follows (Table 3).

**Table 3:** Data-driven loss function matrix.

	$\mathrm{X}$	${\mathrm{X}}^{\mathrm{c}}$
${\mathrm{a}}_{\mathrm{p}}$	${\uplambda }_{\mathrm{PP}}=0$	${\uplambda }_{\mathrm{PN}}={\mathrm{S}}^{\mathrm{c}}(\mathrm{X}|[\mathrm{x}])$
${\mathrm{a}}_{\mathrm{B}}$	${\uplambda }_{\mathrm{BP}}=\mathrm{S}(\mathrm{X}|[\mathrm{x}])(\mathrm{P}(\mathrm{X}|[\mathrm{x}])-\upzeta )$	${\uplambda }_{\mathrm{BN}}={\mathrm{S}}^{\mathrm{c}}(\mathrm{X}|[\mathrm{x}])(1-\mathrm{P}(\mathrm{X}|[\mathrm{x}])-\upzeta )$
${\mathrm{a}}_{\mathrm{N}}$	${\uplambda }_{\mathrm{NP}}=\mathrm{S}(\mathrm{X}|[\mathrm{x}])$	${\uplambda }_{\mathrm{NN}}=0$

Take the questionnaire data of enterprise managers in this paper as an example, all questionnaires of enterprise managers are U (the universe of discourse), and we can take the AA1 index as the decision attribute set, AA11, AA12, and AA13 as the conditional attribute set. The positive domain, negative domain, and decision risk of each conditional attribute set were calculated based on the questionnaire survey data. According to the principle that the larger the positive domain is, the larger the weight is; the larger the negative domain is, the smaller the weight is; the larger the decision risk is, the smaller the weight is, the three attribute weights of W_POS_, W_neg_ and W_risk_ were calculated by using the rough set size of positive domain, negative domain, and decision risk. Based on the related literature^39^, this study employed the positive domain, negative domain, and the risk of the rough set to calculate the three attribute weights of W_pos_, W_neg_, and W_risk_, respectively.

(1) Weight based on the size of the positive domain, Wpos.

For the attribute set $C=\{{a}_{1},{a}_{2},\cdots ,{a}_{i},\cdots {a}_{k}\}$, the weight of the attribute ${a}_{i}$(${x}_{j}\in {a}_{i}$) can be expressed as:

7 $$\widetilde{w}_{i}^{pos} = \exp (\frac{{|x_{j} \in POS_{i}^{S} |}}{|U|})$$

After the normalization, the following expression can be obtained:

8 $$w_{i}^{pos} = \widetilde{W}_{i}^{pos} /\sum\limits_{i = 1}^{k} {\widetilde{W}_{i}^{pos} }$$

Then, the weights of all attributes can be expressed as:

9 $$W_{pos} = \left\{ {W_{1}^{pos} } \right.,W_{2}^{pos} ,...,W_{i}^{POS} ,...,W_{K}^{pos} \left. {} \right\}$$

(2) Weight based on the size of the negative domain, W_neg_.

The weight based on the negative domain can be expressed as:

10 $$\widetilde{W}_{i}^{neg} = \exp (\frac{{|x_{j} \in NEG_{i}^{S} |}}{|U|})$$

Similarly, after the normalization, the following expression can be obtained:

11 $$W_{i}^{neg} = \widetilde{W}_{i}^{neg} /\sum\limits_{i = 1}^{k} {\widetilde{W}_{i}^{neg} }$$

Then, the weights of all attributes can be written as:

12 $$W_{neg} = \left\{ {W_{1}^{neg} ,W_{2}^{neg} ,...,W_{i}^{neg} ,...,W_{k}^{neg} } \right\}$$

(3) Weight based on the Risk, W_risk_.

For the attribute set $\mathrm{C}=\{{\mathrm{a}}_{1},{\mathrm{a}}_{2},\cdots ,{\mathrm{a}}_{\mathrm{i}},\cdots {\mathrm{a}}_{\mathrm{k}}\}$, the risk of the attribute ${\mathrm{a}}_{\mathrm{i}}$(${\mathrm{x}}_{\mathrm{j}}\in {\mathrm{a}}_{\mathrm{i}})$ can be expressed as:

13 $${\mathcal{R}}_{\mathrm{ai}}={\sum }_{\mathrm{x}\in {\mathrm{POS}}^{\mathrm{s}}}\left(1-\mathrm{P}(\mathrm{X}|[\mathrm{x}{]}_{\mathrm{ai}})\right)\cdot {\uplambda }_{\mathrm{PN}}+{\sum }_{\mathrm{x}\in {\mathrm{BND}}^{\mathrm{S}}}\left(\mathrm{P}(\mathrm{X}|[\mathrm{x}{]}_{\mathrm{ai}})\cdot {\uplambda }_{\mathrm{BP}}+(1-\mathrm{P}(\mathrm{X}|[\mathrm{x}{]}_{\mathrm{ai}}))\cdot {\uplambda }_{\mathrm{BN}}\right)+{\sum }_{\mathrm{x}\in {\mathrm{NEG}}^{\mathrm{S}}}\mathrm{P}(\mathrm{X}|[\mathrm{x}{]}_{\mathrm{ai}})\cdot {\uplambda }_{\mathrm{NP}}$$

After the normalization, the following expression can be obtained:

14 $${\overline{\mathcal{R}} }_{\mathrm{ai}}=\frac{{\mathcal{R}}_{\mathrm{ai}}}{{\sum }_{\mathrm{ai}}{\mathcal{R}}_{\mathrm{ai}}}$$

Based on the above risks, the conversion towards the weight can be expressed as:

15 $${\widetilde{\mathrm{w}}}_{\mathrm{ai}}^{\mathrm{risk}}=1-{\overline{\mathcal{R}} }_{\mathrm{ai}}$$

Therefore, the weight of the attribute ${\mathrm{a}}_{\mathrm{i}}$ can be expressed as:

16 $$W_{ai}^{risk} = \frac{{\widetilde{W}_{ai}^{risk} }}{{\sum\nolimits_{ai} {\widetilde{W}}_{ai}^{risk} }}$$

Then, the weights of all attributes can be expressed as:

17 $$W_{risk} = \left\{ {W_{1}^{risk} ,W_{2}^{risk} ,...,W_{i}^{risk} ,...,W_{k}^{risk} } \right\}$$

#### Comprehensive weight calculation method—information entropy and rough set based on Euclidean distance

Based on previous research results of Li et al.^40^ and Suo et al.^39^, this study combined the Euclidean distance, information entropy, and rough set and innovatively proposed a comprehensive weight calculation method based on the three methods. The detailed procedures are described below. Taking the calculation of the importance of the AA1 index entropy weight method as an example, first, calculate the Euclidean distance of the five weights of the AA1 index, and then calculate the information entropy of the five Euclidean distances, denoted as *H*(A). Following a similar approach, the information entropy of the AA1 index is calculated for the four weighting methods Euclidean distance in addition to the entropy method, denoted as *H*(*A* – {*a*_i_}). Calculate the difference between *H*(A) and *H*(*A* – {*a*_i_}) from the perspective of the rough set, which is the importance of the entropy weight method. According to the importance of each index, the comprehensive weight of the AA1 index can be obtained.

The entropy weight method, CRITIC method, and the Pos, Neg, and Risk methods of the rough set were used to calculate the objective weights of the indexes. The method set is denoted as $A = \left\{ {\left. {a_{1} ,a_{2} ,a_{3} ,a_{4} ,a_{5} } \right\}} \right.$, where $a_{i}$ denotes the i-th weight calculation method.

The information entropy of the method set can be expressed as:

18 $$H\left( {\text{A}} \right) = - \sum\limits_{i}^{5} {P\left( {{\text{a}}_{i} } \right)} \ln P\left( {{\text{a}}_{i} } \right)$$

where $P\left( {{\text{a}}_{i} } \right)$ is the related Euclidean distance between various attribute weights.

The importance degree of a single method in the method set A can be expressed as:

19 $$S\left( {a_{{\text{i}}} } \right) = abs\left( {H\left( A \right) - H\left( {A - \left\{ {\left. {a{}_{{\text{i}}}} \right\}} \right.} \right)} \right)$$

where abs(x) denotes the absolute value of x. After the normalization, the weight of a single method can be obtained as:

20 $$\eta_{i} = \frac{{S\left( {a_{i} } \right)}}{{\sum\limits_{k = 1}^{5} {S\left( {a_{k} } \right)} }}\quad (i = 1,2,3,4,5)$$

The comprehensive weight can be determined as:

21 $${\text{W}} = \sum\limits_{i = 1}^{5} {\eta_{i} y_{i} }$$

where W denotes the comprehensive weight based on the rough set and the information entropy, and y_i_ denotes the weight of the i-th method. The “final weight W” in Fig. 5 is the weight of the index at its corresponding level.

### Data collection and organization

#### Expert scoring data

This paper invites 5 experts to rate the importance of subsystems and first-level indicators in the risk evaluation system. The youngest of the 6 experts is 43 years old, and the oldest is 62 years old. They all have senior professional titles and have been engaged in safety management and safety evaluation in the coal mine field for more than 20 years. They have a long-standing business and working exchanges with government regulatory departments and provincial coal mining enterprises. They participate in research projects, conduct safety inspections at the front line of coal mines, and provide advice to the government in formulating regulatory policies, and are familiar with the current situation and safety risks faced by the coal mining industry in province A. Their scoring data are highly representative and authoritative. The detailed data of experts' scores are shown in Online Appendix B.

#### Survey data

This paper designs 89 Likert scales for a total of 90 factor indexes at the first, second, and third levels (BB1 is the same as BB and does not include the first-level indicators of subsystem E). Moreover, the scale questions are put into three types of questionnaires (questionnaire I is shown in Online Appendix A). As a staff member of the provincial regulatory department, the author took advantage of the job opportunity to conduct a three-month interview with government supervisors, government-employed experts (questionnaire I), coal mine enterprise administrators (questionnaire II), and front-line workers (questionnaire III) in province A. A questionnaire survey was conducted and a total of 196 questionnaires were distributed, among which 189 questionnaires were recovered and 170 were effective. The recovery rate of effective questionnaires was 86.7%. Tables 4 and 5 show the releasing condition of questionnaires. In order to avoid misgivings in filling out questionnaires (for example, some enterprise administrators answered the question regarding the overmanned underground operation (AC24) of the enterprise), some sensitive indexes (AA11-AA85) were simultaneously put into two types of questionnaires. The purpose is to expand the data source range, and compare the same types of data from different sources, thereby eliminating the system error in the questionnaire survey as much as possible.

**Table 4:** Number of participants in questionnaire I.

Department level	Total staff	Participants in the questionnaire survey	Survey ratio	Types of questionnaires and the secondary indicators included
Provincial coal mine supervision department	5	5	100%	Questionnaire I, BA1-BA8,BB1,BC1-BC2, AA1-AA4,AA7-AA8
Municipal coal mine supervision department	8	6	75%	
County-level coal mine supervision department	14	12	85.7%	
Experts hired by supervision department	19	15	78.9%

**Table 5:** Number of participants in questionnaire II, III.

Types of jobs	Enterprise A	Enterprise B	Enterprise C	Enterprise D	Participants in the questionnaire survey	Survey ratio	Types of questionnaires and the secondary indicators included
Enterprise management	13	10	7	10	–	–	Questionnaire II, AA1-AA8, AB1-AB3,AC1-AC3
Enterprise management responsible for safety management	11	7	6	7	28	90.3%	
Enterprise mid-level management	33	25	19	16	24	25.8%	
Other managers	69	45	25	25	29	17.7%	
Coal mining front-line staff	135	130	166	144	18	3.13%	Questionnaire III, CA1-CA6, CB1-CB2, CC1-CC3, AA5-AA6
Tunneling front-line staff	248	102	74	55	19	3.97%	
Electromechanical-transport staff	196	148	54	47	16	3.59%	
Staff responsible for “One Ventilation and Three Prevention”	254	133	16	7	16	3.9%	
Other underground staff	480	130	54	75	3	0.4%	

In this survey, over 75% of supervisors at all levels were involved in the present questionnaire survey and over 90% of administrators at the enterprise management level were investigated. The survey also covered almost all types of jobs in underground mines. Because 62.3% of the front-line workers in coal mines in province A have junior high school education or below (many of them may only have a primary school education or are illiterate), many workers cannot accurately evaluate their own safety concepts and safety skills. To ensure the accuracy and effectiveness of the data, we only invite the squad leaders or team leaders of the front-line workers to participate in the survey. Some team leaders are unwilling to participate in the survey for some reasons, which leads to a low survey coverage of front-line workers. But this coverage is the best we can do. Considering that enterprise managers have a significant impact on the safety status of front-line workers, the extremely high coverage of managers can partially offset the impact of low coverage of front-line employees, so the low questionnaire participation rate of front-line workers is acceptable in this study. Through calculation, the Cronbach α coefficients of questionnaires I, II, and III were 0.9, 0.932, and 0.851, respectively, indicating that the reliability of this questionnaire is high, the real response rate of the questionnaire participants is high, and the coverage is extremely wide and representative.

Validity is used to measure whether the item (quantitative data) design is reasonable. Table 6 lists the KMO values of the questionnaire. The KMO values of 26 scale questions at the government regulation level (subsystem B) in the questionnaire I were 0.518 and lower than 0.6, suggesting poor measure performance. Considering the low frequency of national regulatory departments inspecting a single mine, its influence on the safety risk of a single enterprise is limited and thus is suggested to remove the BA11 index from the system. After removal, the KMO value in validity analysis was 0.678, satisfying the requirement in scale design.

**Table 6:** Number of participants in the whole questionnaire.

Types of questionnaires	Quantities of questionnaire	Subsystems	Number of scale questions involved	KMO value
Questionnaire I	35	Government regulatory level (B)	26	0.518
Questionnaire I (modified)	35	Government regulatory level (B)	25	0.678
Questionnaire II	73	Enterprise management level (A)	49	0.719
Questionnaire III	62	Front-line workers level (C)	14	0.632

### Weight calculation process

**Figure 4:**
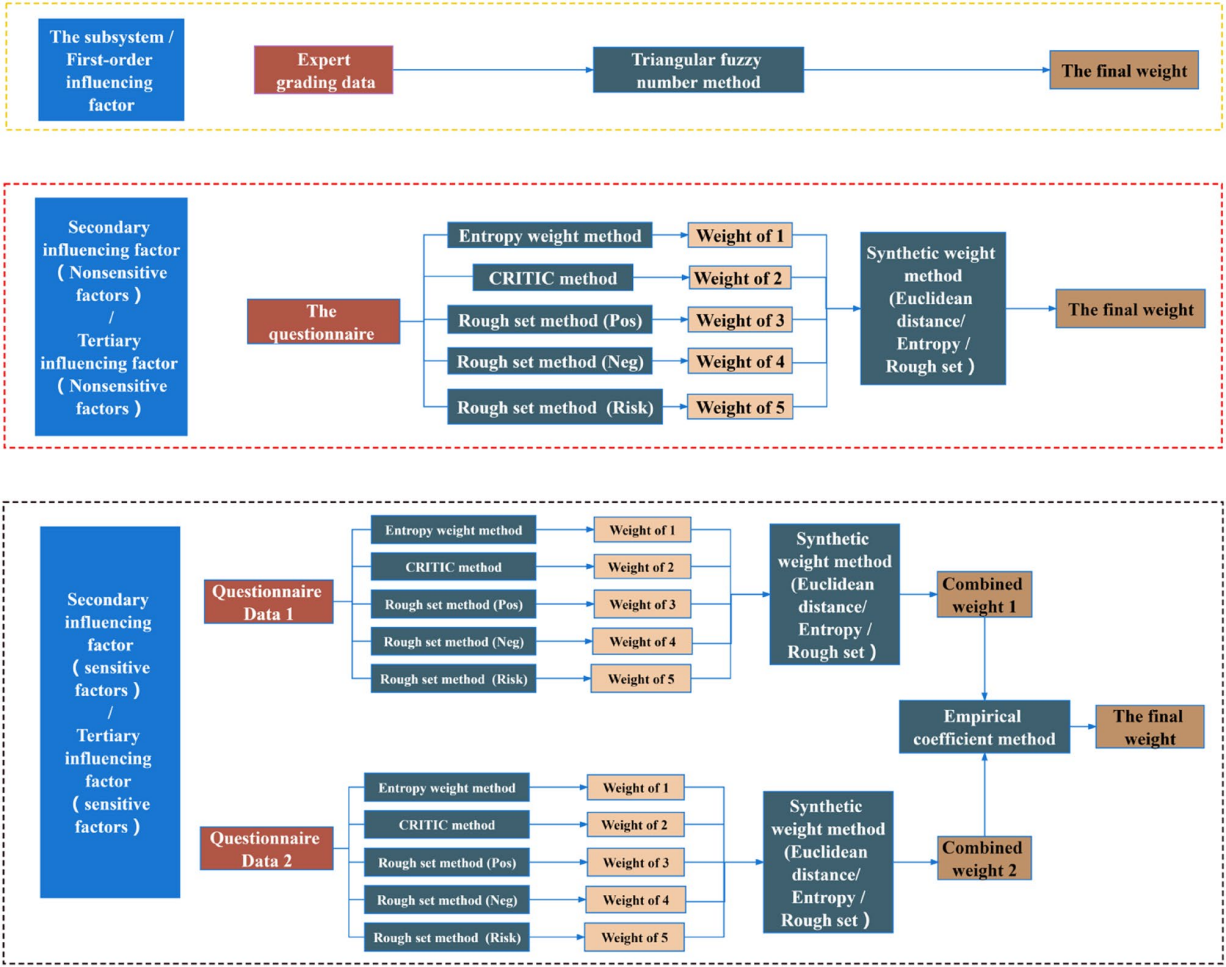
Weight calculation method diagram of indexes at all levels.

**Figure 5:**
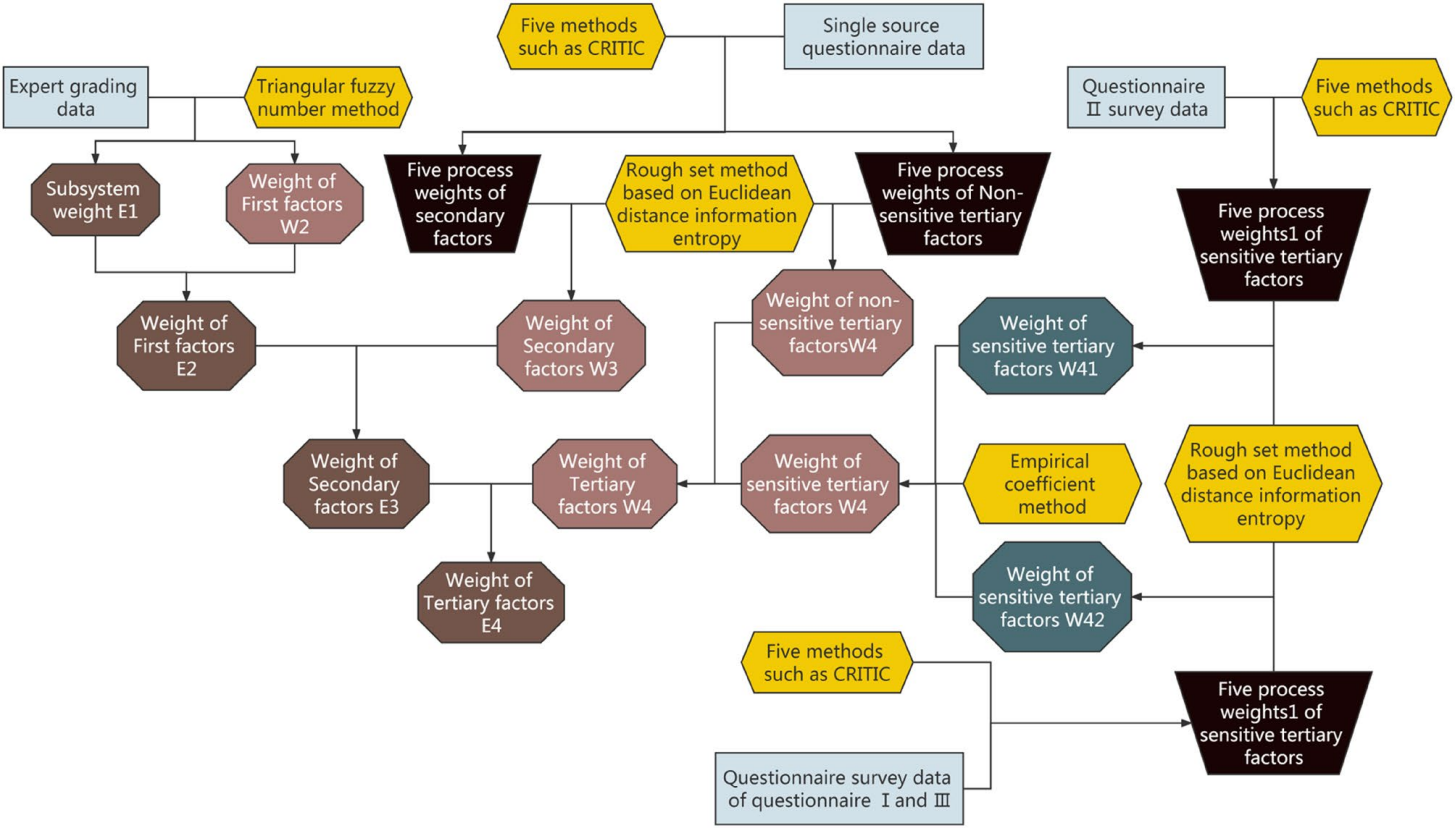
Flow chart of weight calculation.

This study used the triangular fuzzy number function to analyze the expert score on the subsystems and the first-level indexes, and then calculated their weights. For the second- and third-level indexes, this study uses five objective weighting methods, i.e., entropy weight method, CRITIC method, the Risk, Pos, and Neg methods of the rough set, to analyze the data of the questionnaires. Then, a comprehensive weighting method combining the information entropy of Euclidean distance and the rough set was used to calculate their comprehensive weights. For the second- and third-level indexes (AA11-AA85) of sensitive factors with multiple data sources, the importance of data sources was taken as the importance of the calculated comprehensive weights, which can merge with a variety of comprehensive weights to obtain the final weight. The calculation process of the weights is displayed in Fig. 4. It should be noted that the final weights mentioned above are the weights of the indicators in the hierarchy, which are W_2_, W_3_ for the primary indicators, W_4_ for the secondary indicators, and W_4_ for the tertiary indicators. Multiplying the primary indicator weight W_2_ by the subsystem weight E_1_ is the weight of the primary indicator in the whole indicator system, which is E_2_, and the calculation process is shown in Fig. 5.

## Results and discussion

### Weight calculation results and subject evaluation results

**Figure 6:**
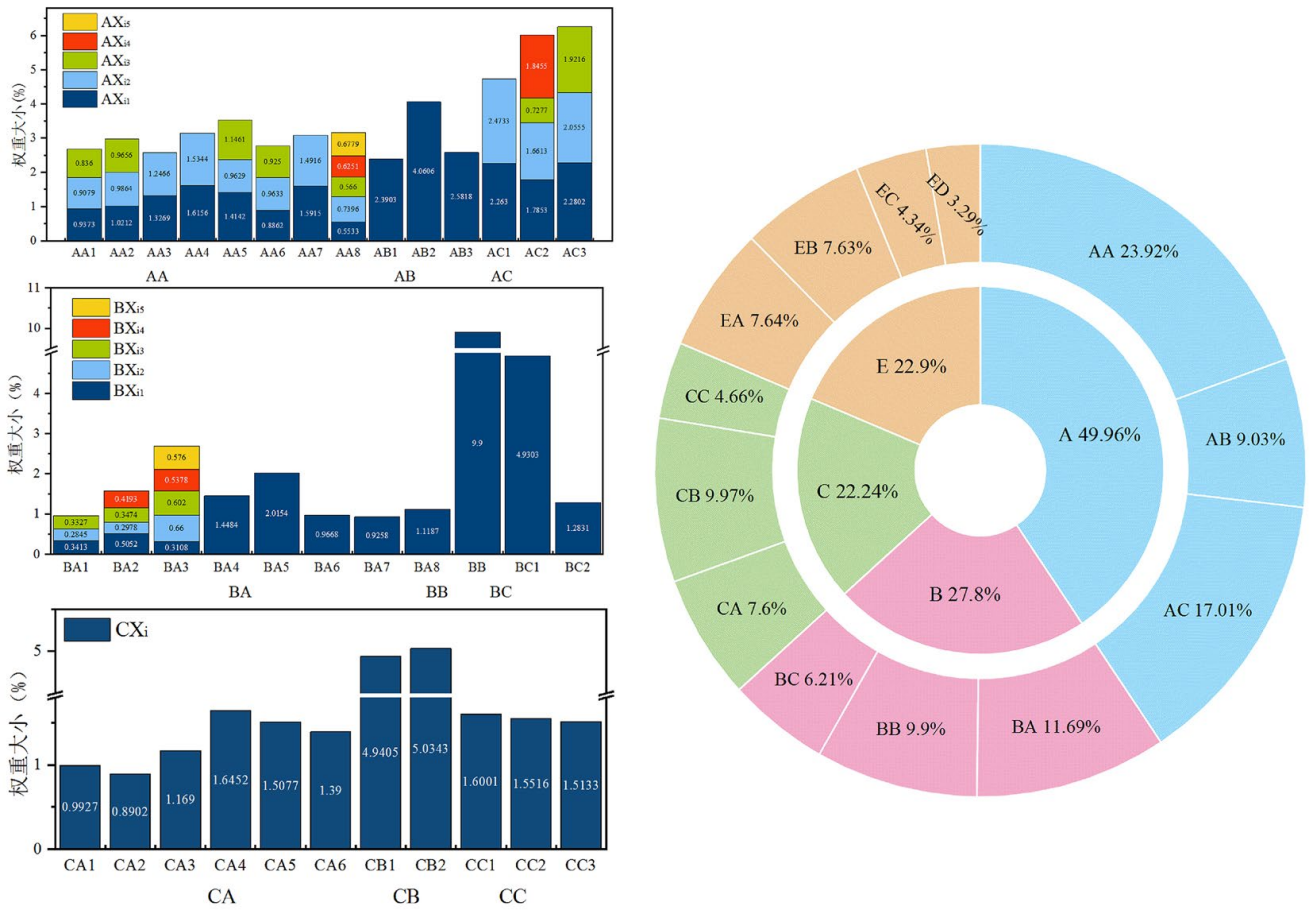
Calculation of weights for each level of the index.

The final results of weight calculation of indicators at each level (E_i_) are shown in Fig. 6 and the results of process weight calculation in the middle (W_i_) are shown in Online Appendix C. From the results, it can be seen that among the four subsystems, Enterprise management level (Subsystem A) has the highest weight of 49.96%, which shows that enterprises fulfill the main responsibility of safety production is the basic condition in doing a good job of safety production; Government regulatory level (Subsystem B) has a weight of 27.8%, which is slightly higher than Front-line workers level (Subsystem B) at 22.24%, indicating that China attaches great importance to government safety supervision; The weight of External factor Level (Subsystem E) is 22.9%, which is higher than that of Subsystems B and C, proving that enterprise managers and government supervisors should pay full attention and concern to the influence of external environment on enterprise safety risks. Within the Enterprise management level (Subsystem A) and Government regulatory level (Subsystem B), Management personnel operating pressure (primary indicator AB) and Safety regulatory economic aspects (primary indicator BB) were usually neglected in the past, but their importance also reached 18.08% (W2) and 35.61% (W_2_), respectively. In the External factor Level (Subsystem E), EA (Ore prices) and EB (Industry policies adjustment) are more important than EC (Production safety accidents occur with great impact) and ED (Underground geological conditions of enterprises have drastic changes).

Combining the questionnaire survey and the results of weight calculation, this paper calculated the safety management personnel, front-line workers, and provincial, municipal, and county-level supervisors of four coal mining enterprises in province A in three subsystem scores of A, B, and C (the higher the score, the lower the risk), and derived the safety risk status of each subject. The scores are shown in Tables 7, 8, and 9. It can be seen that the safety risk in all three dimensions of the safety management level of A and B coal mines is greater than that of C and D. The supervision effect of the supervisory department is greater at the provincial level than at the municipal level, and at the municipal level than at the county level. The safety status of front-line workers in D mine is better than that of A and C. Because B mine was out of production at the time of the questionnaire survey, only the managers of B mine were surveyed, without investigating its front-line workers.

**Table 7:** Score ranking of safety management work (subsystem A) of four enterprises.

Enterprise management level	Enterprise A		Enterprise B		Enterprise C		Enterprise D	
	Score	Ranking	Score	Ranking	Score	Ranking	Score	Ranking
AA working level	67.11	4	69.59	3	75.91	2	75.95	1
AB pressure level	59.63	4	62.11	3	62.93	2	62.98	1
AC economic level	59.97	3	58.82	4	70.45	1	61.58	2
Subsystem A total score	63.32	4	64.56	3	71.71	1	68.71	2

**Table 8:** Score ranking of supervision effect (subsystem B) of three-level supervision departments in provinces, cities, and counties.

Government regulatory level	Provincial level		Municipal level		County level	
	Score	Ranking	Score	Ranking	Score	Ranking
BA regulatory outcomes	67.81	2	72.71	1	60.19	3
BB regulatory economic level	92	1	70	3	85	2
BC regulatory pressure	70.48	1	61.19	2	50	3
Subsystem B total score	77.03	1	69.18	2	66.76	3

**Table 9:** Ranking of front-line employee safety status (subsystem C) scores of the three companies.

Enterprise front-line employee level	Enterprise A		Enterprise B		Enterprise C	
	Score	Ranking	Score	Ranking	Score	Ranking
CA Safety atmosphere	71.09	2	70.94	3	75.86	1
CB Should know and master	81.27	2	76.57	3	82.89	1
CC Worker Safety Status	68.62	3	71.55	2	82.84	1
Subsystem C Total Score	75.14	2	73.59	3	80.78	1

### Performance validation of the established evaluation system

The safety risk evaluation system in this paper contains 98 factor indexes. Considering the influence of the external environment such as coal price and industry policy changes on risks, no similar evaluation system can be found in the existing literature for comparison. In order to verify the scientificity, rationality, and reliability of the evaluation system, this paper compares the weighted scores of the indicators of different subjects with the real data of the subject, and analyzes whether the weighted scores can represent the changing trend of the real data, so as to verify the safety risk assessment system in this paper. Due to the limitation of real data types and data volume, only some conditional and representative indicators can be selected for comparison.

#### Verification of evaluation results of some indicators in subsystem A

The standardized management system of the safety production of coal mines is a set of management systems for static evaluation of the safety production condition of enterprises by the government departments, which is the most comprehensive safety management evaluation system for coal mining enterprises in China. It includes 9 major categories, 173 minor categories, and nearly 600 inspection items. After the enterprise passes through the self-evaluation, the supervision departments hire 8–10 professional staff with over 10 years of working experience to conduct a comprehensive system inspection, list the existing hidden problems, and score these items. Based on the weights, the final score of the enterprise can be obtained. Based on the weighted score, the enterprises are classified into Level 1, Level 2, and Level 3, and the government does not allow normal production of coal mining enterprises that have not obtained Level 3 or higher standardization. This section makes a comparision between the total scores of the standardized management system assessment of the three coal mines in province A and the weighted scores of "AA enterprise safety management work quality", between the scores of continuous improvement of the standardized management system assessment and the weighted scores of "AA8 feedback summary and improvement intensity". In order to distinguish the evaluation discrimination degree of each evaluation system, the discrimination degree of the evaluation system is calculated, which is denoted as Ψ. Taking the weighted score of the AA index as an example, the average weighted score of the AA index of the three coal mines A, C, and D is calculated as 72.99, and then the difference between the scores of the three coal mines and 72.99 is calculated, which are -5.88 and 2.92, and 2.96, respectively. Then we divide the difference of each enterprise by the average, take the absolute value and express the weighted score as a percentage. The weighted score and Ψ calculation results are shown in the Tables 10 and 11.

**Table 10:** Results of the total standardized management system score compared to the weighted score of AA indicators.

	Enterprise A		Enterprise C		Enterprise D	
	Score	Ψ value (%)	Score	Ψ value (%)	Score	Ψ value (%)
Weighted score of AA indicators	67.11	8.06	75.91	4	75.95	4.06
Standardized assessment scores in 2020	77.1	4.48	81.03	0.38	84.03	4.1
Standardized assessment scores in 2021	83.29	0.24	83.69	0.24	–

**Table 11:** Results of the continuous improvement component of the standardized management system compared to the weighted AA8 indicator scores.

	Enterprise A		Enterprise C		Enterprise D	
	Score	Ψ value (%)	Score	Ψ value (%)	Score	Ψ value (%)
Weighted score of AA indicators	67.11	8.06	75.91	4	75.95	4.06
Scores for the "continuous improvement" part of the standardized assessment in 2020	77.1	4.48	81.03	0.38	84.03	4.1
Scores for the "continuous improvement" part of the standardized assessment in 2021	83.29	0.24	83.69	0.24	–	

It can be seen that the weighted scores of the AA indicators of different enterprises are consistent with the scores and trends of the two standardized assessments, and the value of the evaluation discrimination degree Ψ of the AA indicator scores is better than that of the standardized management system. The evaluation discrimination degree of the AA8 index score is also better than the standardized evaluation "continuous improvement" evaluation. It proves that the safety risk evaluation system in this paper is scientific and effective, and its degree of discrimination is better than that of the coal mine safety production standardization management system.

#### Verification of the evaluation results of the indicators of Part B of the subsystem

This paper collects the educational background and professional status of all supervisory personnel of the three-level supervisory department in province A, calculates the average number of hidden dangers found in a single inspection by the three-level supervisory department in 2020, and compares it with the BA3 supervisory ability score. The results verify the rationality and accuracy of the use of this indicator to evaluate the supervision capabilities of supervisors at all levels (Table 12).

**Table 12:** Comparison of the supervision effect score of provincial, municipal, and county-level supervision departments (subsystem B) and the basic information of supervisors.

	BA3 supervision capability score	Education background		Proportion of graduates majoring coal mines (%)	Average number of hidden dangers found in a single inspection
		Ratio of master (%)	Ratio of bachelor (%)		
Provincial	67.92	40	60	100	20.3
Municipal	65.25	15.79	42.1	63.16	15.6
County-level	49.58	0	20	75	4.2

#### Verification of the evaluation results of indicators in subsystem C

The maximum monthly income data of front-line workers of three coal mines in province A were collected and compared with the score of the income satisfaction degree (CA4). On that basis, the reasonability and accuracy of the index in evaluating the income satisfaction degree by enterprise staff were validated (Table 13).

**Table 13:** Comparison of the safety state score (subsystem C) of front-line workers and the average monthly income of coal miners in three enterprises.

	CA4 Income satisfaction score	Average monthly income/RMB
Enterprise A	53.76	6345
Enterprise B	60.63	8524
Enterprise C	64.29	9386

### Horizontal and longitudinal comparisons of the evaluation results

#### Horizontal comparisons of the evaluation results

Compare the score of underlying factor index in a subject with the average score of the same factor index of the same type of subject. For example, compare the score of the hidden danger screening quality (AA21) of A enterprise with the average score of the hidden danger screening quality (AA21) of A, B, C, and D enterprises, and then check whether the hidden danger screening quality (AA21) of A enterprise has reached the average work quality of the same type enterprises in the same region. If the horizontal comparison score is positive, it indicates that the hidden danger screening quality (AA21) of A enterprise exceeds the average level of the same type of local enterprises; If the score is negative, the hidden danger screening quality (AA21) of A enterprise is lower than the average level of the same type local enterprises. It should be noted that there are two horizontal comparison results in this study. First, the original score of the factor index questionnaire is used for calculation, comparison, and analysis, which is called the horizontal comparison of original scores; Second, by multiplying the original score of the factor index questionnaire by the final weight of the factor index, and then using the obtained weight score for calculation, comparison, and analysis, which is called the horizontal comparison of weight scores. The horizontal comparison of the original scores is to compare the scores of the work reflected by various index factors from the perspective of participants in the questionnaire survey. The horizontal comparison of the weight scores after adding the index weights incorporates the preferences of the questionnaire participants and the risk evaluation system designers, which can better reflect the concerns of the designers.

In order to find out the underlying work in an evaluation subject that needs to be improved most, we assume $\alpha_{k}$ is the index with the largest difference in original scores, and $\beta_{k}$ is the index with the largest difference in weight scores. They can be calculated as follows:

22 $$\alpha_{k} = \min \left[ {\left( {x_{k1} - \overline{{x_{i1} }} } \right),\left( {x_{k2} - \overline{{x_{i2} }} } \right), \cdots ,\left( {x_{kj} - \overline{{x_{ij} }} } \right)} \right]$$

23 $$\beta_{k} = \min \left[ {\omega_{1} \left( {x_{k1} - \overline{{x_{i1} }} } \right),\omega_{2} \left( {x_{k2} - \overline{{x_{i2} }} } \right), \cdots ,\omega_{j} \left( {x_{kj} - \overline{{x_{ij} }} } \right)} \right]$$

where $x_{ij}$ denotes the score of the j-th index in the i-th sample, $\overline{{x_{ij} }}$ denotes the average score of the j-th index in all i-th samples, and $\omega_{j}$ denotes the weight of the j-th index.

Tables 14, 15, 16 and 17 show the horizontal comparison results of the safety management (AA of the first-level indexes), management level (subsystem A), and front-line workers (subsystem C) of different enterprises, and the supervision level of the supervision departments at provincial, municipal, and county levels (subsystem B).

**Table 14:** Analysis of the shortcomings of the basic index scores of the safety management in four companies (AA of the first-level indexes)—horizontal comparison.

Horizontal comparison of AA of the four enterprises	Original score		Weight score	
	Factor index	Gap/α	Factor index	Gap/β
Enterprise A	AA31 Completeness of enterprise regulations and policies	− 0.5729	AA31 Completeness of enterprise regulations and policies	− 0.0076
Enterprise B	AA52 Punishments	− 0.4288	AA52 Punishments	− 0.0041
Enterprise C	AA81 In-time identification of common problems	− 0.0891	AA41 Quality of management personnel	− 0.0008
Enterprise D	AA53 Rewards	0.0119	AA53 Rewards	0.0001

**Table 15:** Analysis of the shortcomings of the basic index scores of the safety management in four companies (subsystem A)—horizontal comparison.

Horizontal comparison of subsystem A of the four enterprises	Original score		Weighted score	
	Factor index	Gap/α	Factor index	Gap/β
Enterprise A	AA31 Completeness of enterprise regulations and policies	− 0.57292	AC11 Input of new technologies	− 0.00947
Enterprise B	AA52 Punishments	− 0.42875	AB1 Corporate profitability pressure	− 0.01181
Enterprise C	AB3 Safety pressure	− 0.15375	AB3 Safety pressure	− 0.00397
Enterprise D	AC24 Impulse of overmanned underground operation	− 0.55375	AC24 Impulse of overmanned underground operation	− 0.01022

**Table 16:** Analysis of the shortcomings of the basic index scores of provincial, municipal and county-level supervisory departments (subsystem B)—horizontal comparison.

Horizontal comparison of supervision departments	Original score		Weight score	
	Factor index	Gap/α	Factor index	Gap/β
Provincial	BA21 Administrative penalty	− 0.75	BA4 Supervision initiative	− 0.0048
Municipal	BB Financial pressure of department operation	− 0.6167	BB Financial pressure of department operation	− 0.0611
County-level	BA32 Quality of supervisory cadres	− 0.9333	BC1 Social stability pressure	− 0.0381

**Table 17:** Analysis of the shortcomings of the basic index scores of front-line workers' safety status (subsystem C) in three companies—horizontal comparison.

Horizontal comparison of indexes in subsystem C of three enterprises	Original score		Weight score	
	Factor index	Gap/α	Factor index	Gap/β
Enterprise A	CC2 Safety awareness of workers	− 1.0818	CC1 Safety concept	− 0.0173
Enterprise C	CB2 Self-management ability	− 0.2649	CB2 Self-management ability	− 0.0133
Enterprise D	CC3 "Three violations" of workers	− 0.1146	CC3 "Three violations" of workers	− 0.0017

It can be seen from Table 14 that the values of $\alpha_{k}$ and $\beta_{k}$ in enterprise D are positive numbers, reflecting that the safety management quality of enterprise D is generally stronger than that of A, B, and C enterprise. There are major deficiencies in the integrity of the company's rules and regulations in enterprise A, the severity of punishment in enterprise B, the quality of safety management personnel in enterprise C, and the discovery of generally hidden dangers. Table 15 shows that enterprise A's system is not perfect, and new technology investment is insufficient, enterprise B's punishment needs to be strengthened, and the pressure on profitability is relatively high, enterprise C's management has serious pressure on safety, and enterprise D's impulse to go underground due to overcrowding is relatively serious. Table 16 shows that provincial supervision departments are not active in law enforcement and administrative penalties are relatively low, municipal supervision departments are under great pressure on economic operation, and county-level supervision departments are under great pressure on social stability and the ability of cadres is difficult to meet the requirements. Table 17 shows that the safety awareness and safety concept of workers in enterprise A, the self-management ability of workers in enterprise C, and the phenomenon of three violations in enterprise D is far from the average level of brother units.

#### Longitudinal comparisons of the evaluation results

A subject’s low score for a certain indicator may be due to the individual’s poor ability and low work enthusiasm, or it may be due to objective factors that limit the score of this indicator. For the latter condition, only by changing some top-level design or environment can this indicator be effectively improved. In order to find out these indicators with low scores due to external objective reasons, this paper compares the score of a underlying factor index in a subject with the average score of all factor indexes of the subject. For example, compare the score of the hidden danger screening quality (AA21) of A enterprise with the average score of all underlying factor indexes (third-level indicators) at the work level of A enterprise (subsystem A), and then check whether the hidden danger screening quality (AA21) of A enterprise has reached the average work quality of all the work of the enterprise. If the longitudinal comparison score is positive, it indicates that the hidden danger screening quality (AA21) of A enterprise exceeds the average work quality of the enterprise; If the score is negative, the hidden danger screening quality (AA21) of A enterprise is lower than the average level of the work quality of the enterprise. It should be noted that there are two longitudinal comparison results in this study. First, the original score of the factor index questionnaire is used for calculation, comparison, and analysis, which is called the longitudinal comparison of original scores; Second, by multiplying the original score of the factor index questionnaire by the final weight of the factor index, and then using the obtained weight score for calculation, comparison, and analysis, which is called the longitudinal comparison of weight scores. The longitudinal comparison of the original scores is to compare the scores of the work reflected by the various index factors from the perspective of participants in the questionnaire survey. The longitudinal comparison of the weight scores after adding the index weights incorporates the preferences of the questionnaire participants and the risk assessment system designers, which can better reflect the concerns of the designers.

In order to find out the underlying work in an evaluation subject that needs to be improved most, we assume $\eta_{k}$ is the index with the largest difference in original scores, and $\lambda_{k}$ is the index with the largest difference in weight scores. They can be calculated as follows:

24 $$\eta_{k} = \min \left[ {\left( {x_{k1} - \overline{{x_{kj} }} } \right),\left( {x_{k2} - \overline{{x_{kj} }} } \right), \cdots ,\left( {x_{kj} - \overline{{x_{kj} }} } \right)} \right]$$

25 $$\lambda_{k} = \min \left[ {\omega_{1} \left( {x_{k1} - \overline{{x_{kj} }} } \right),\omega_{2} \left( {x_{k2} - \overline{{x_{kj} }} } \right), \cdots ,\omega_{j} \left( {x_{kj} - \overline{{x_{kj} }} } \right)} \right]$$

where $x_{ij}$ denotes the score of the j-th index in the i-th sample, $\overline{{x_{1j} }}$ denotes the average score of all indexes in the first sample, and $\omega_{j}$ denotes the weight of the j-th index.

Tables 18, 19, 20 and 21 show the longitudinal comparison results of the safety management (AA of the first-level indexes), management level (subsystem A), and front-line workers (subsystem C) of different enterprises, and the supervision level of the supervision departments at provincial, municipal, and county levels (subsystem B).

**Table 18:** Analysis of the shortcomings of the basic index scores of the safety management in four companies (AA of the first-level indexes)—longitudinal comparison.

Longitudinal comparison of AA of the four enterprises	Original score		Weight score	
	Factor index	Gap/η	Factor index	Gap/λ
Enterprise A	AA53 Rewards	− 0.6304	AA72 Site construction quality	− 0.0094
Enterprise B	AA52 Punishments	− 0.8043	AA72 Site construction quality	− 0.0094
Enterprise C	AA72 Site construction quality	− 0.2661	AA72 Site construction quality	− 0.0041
Enterprise D	AA52 Punishments	− 0.7791	AA53 Rewards	− 0.0089

**Table 19:** Analysis of the shortcomings of the basic index scores of the safety management (subsystem A) in four companies—longitudinal comparison.

Longitudinal comparison of the indexes of subsystem A of the four enterprises	Original score		Weight score	
	Factor index	Gap/η	Factor index	Gap/λ
Enterprise A	AB1 Corporate profitability pressure	− 1.03214	AB1 Corporate profitability pressure	− 0.04191
Enterprise B	AB1 Corporate profitability pressure	− 1.29286	AB1 Corporate profitability pressure	− 0.05251
Enterprise C	AB1 Corporate profitability pressure	− 1.07886	AB1 Corporate profitability pressure	− 0.04381
Enterprise D	AB1 Corporate profitability pressure	− 1.08691	AB1 Corporate profitability pressure	− 0.04413

**Table 20:** Analysis of the shortcomings of the basic index scores of provincial, municipal and county-level supervisory departments (subsystem B)—longitudinal comparison.

Longitudinal comparison of supervision departments	Original score		Weight score	
	Factor index	Gap/η	Factor index	Gap/λ
Provincial	BA21 Administrative penalty	− 1.45	BA21 Administrative penalty	− 0.0073
Municipal	BC1 Social stability pressure	− 0.8265	BC1 Social stability pressure	− 0.0425
County-level	BC1 Social stability pressure	− 0.9816	BB Financial pressure of department operation	− 0.0651

**Table 21:** Analysis of the shortcomings of the basic index scores of front-line workers' safety status (subsystem C) in three companies—horizontal comparison.

Longitudinal comparison of indexes in subsystem C of three enterprises	Original score		Weight score	
	Factor index	Gap/η	Factor index	Gap/λ
Enterprise A	CC1 Safety concept	− 1.8409	CC1 Safety concept	− 0.0295
Enterprise C	CC1 Safety concept	− 0.9063	CC1 Safety concept	− 0.0145
Enterprise D	CA4, CA5, CA6	− 0.8377	CA4 Income satisfaction degree	− 0.0138

Tables 18, 19, 20 and 21 show that the profit pressure of the four coal mining enterprises in province A has generally increased, the quality of on-site construction management is poor, the rewards and punishments are insufficient, and the safety concept and job satisfaction of front-line workers are very low. The provincial regulatory authorities do not have high administrative penalties, the municipal and county-level regulatory authorities have high pressure on social stability, and the county-level departments have high pressure on the economic operation. It may be that some external objective factors lead to higher scores for these tasks. Only by changing some top-level design or external aspects can this work be effectively improved, and this requires the focus of the superior enterprise of the coal mine and the central government.

## Conclusion

1. The coal mine safety risk perception system established in this paper includes 98 index factors in multiple dimensions such as work quality, pressure, and economy. The calculation results of index weights show that enterprises fulfilling the main responsibility of safety production is the basic condition for doing a good job in safety production work; the performance of safety supervision duties by government departments is of great significance to reduce enterprise safety risks. Management personnel operating pressure (first-level indicator AB) and safety regulatory economic aspects (first-level indicator BB) are very important for good safety work. In the current difficult environment, enterprise managers and government supervisors should pay full attention and focus on the impact of the external environment on enterprise safety risks, where EA (Ore prices), and EB (Industry policies adjustment) are more important than EC (Production safety accident occur with great impact) and ED (Underground).
2. The evaluation results of the safety risk system coincide with the assessment of the coal mine safety production standardization management system and other existing data, and the safety risk system has a good evaluation effect and can perceive a higher dimensional coal mine enterprise safety risk situation. The evaluation results show that the safety management quality of mine D (first-level index AA) is generally stronger than that of companies A, B, and C. On the level of enterprise safety management (subsystem A), the safety risks of mines C and D are lower than those of mines A and B. The safety status of front-line workers (subsystem C) in mine D is better than A and C. In terms of regulatory effect (subsystem B), the provincial level is greater than the municipal level, and the municipal level is greater than the county level.
3. The administrative penalties imposed by the provincial supervision departments are not high, the social stability pressure of the municipal and county-level supervision departments is relatively high, and the economic operation pressure of the county-level departments is high. These aspects have a great impact on the effect of government supervision. It is recommended that the central government and top-level policy designers, strengthen financial support to reduce economic pressure on county-level regulatory departments, strengthen incentive mechanisms to enhance administrative penalties for provincial regulatory departments, pay close attention to the employment situation of personnel in cities with depleted resources, and reduce the pressure on social stability brought about by the implementation of policies to eliminate backward production capacity to safety regulatory departments.
4. Coal mine safety risk management is a systematic project and an interconnected organic whole, involving multiple subjects such as enterprise management, enterprise employees, government regulators, and the external environment. If any of them has a problem, the safety risk of the enterprise will be greatly increased. Limited by the lack of capacity and data, this paper does not quantify the impact of the external environment, especially coal price and policy changes on coal mine safety risk, and much work needs to be done in the future.

### Ethical approval

We declare that we did not submit our manuscript to a preprint server before submitting it to Scientific Reports.

## Supplementary Information

Supplementary Information.

## Data Availability

The datasets used and/or analysed during the current study available from the corresponding author on reasonable request.
