# Supplementary material for: Multi-dimensional safety risk assessment on coal mines under the profitability dilemma
Source: Sci Rep. 2023 Feb 15;13:2687. doi: 10.1038/s41598-023-29795-3 (PMC9931701; doi:10.1038/s41598-023-29795-3)
Supplement: Supplementary file 1 — Supplementary Information. [file 41598_2023_29795_MOESM1_ESM.docx]

Appendix A

**Questionnaire for government supervisors (experts hired by supervision department)**

**Questionnaire I**

1. What is your age group？

| ○35 and below | ○36~40 | ○41~45 | ○46~50 | ○51~55 | ○56~60 |  |
| --- | --- | --- | --- | --- | --- | --- |

2. What is your education？

| ○Junior high school and below | ○High School | ○Associate degree | ○Undergraduate | ○Master and above |
| --- | --- | --- | --- | --- |

3. What is your major？

| ○Mining subject specialties (mining, ventilation, electromechanics, geology, etc.) |
| --- |
| ○Other engineering disciplines |
| ○Physics |
| ○Management Studies |
| ○Other |

4. What is the level of the department you work in?

| ○Provincial coal mine supervision department | ○Municipal coal mine supervision department | ○County-level coal mine supervision department | ○Experts hired by supervision department |
| --- | --- | --- | --- |

5. How long have you been working in coal mining-related jobs?

| ○ Within 3 years | ○3~5 years | ○5 to 10 years | ○10 to 20 years | ○20 years or more |
| --- | --- | --- | --- | --- |

6. How long have you been in your current position?

| ○ Within 1 year | ○1 year~3 years | ○3~5 years | ○5 to 10 years | ○10 years or more |
| --- | --- | --- | --- | --- |

7. To the best of your knowledge, please rate the frequency of inspections of coal mines in your jurisdiction by the regulatory authorities at all levels

|  | Low | Relatively Low | Middle | Relatively High | High |
| --- | --- | --- | --- | --- | --- |
| National level | ○ | ○ | ○ | ○ | ○ |
| Provincial level | ○ | ○ | ○ | ○ | ○ |
| Municipal level | ○ | ○ | ○ | ○ | ○ |
| County level | ○ | ○ | ○ | ○ | ○ |

8. Are you under pressure in your supervisory role?

| ○No pressure | ○Nearly stress-free | ○No feeling | ○A little bit pressure | ○High pressure |
| --- | --- | --- | --- | --- |

9. Please evaluate the following pressures at this level of supervision

|  | Low | Relatively Low | Middle | High | Very High |
| --- | --- | --- | --- | --- | --- |
| Pressure to hold accountable for accidents | ○ | ○ | ○ | ○ | ○ |
| Checking the pressure of fines for the proper functioning of the department | ○ | ○ | ○ | ○ | ○ |
| Social stability pressures on miners with nowhere to earn a living after the closure of coal mines | ○ | ○ | ○ | ○ | ○ |

10. Please rate your own department's proactivity in regulating and enforcing penalties

| ○Very high motivation |
| --- |
| ○Highly motivated |
| ○Generally motivated |
| ○Less motivated |
| ○Poor motivation |

11. Please evaluate the following questions in the light of the actual situation

|  | Very bad | Not good | General | Good | Very good |
| --- | --- | --- | --- | --- | --- |
| Your own supervision capacity | ○ | ○ | ○ | ○ | ○ |
| Your own basic qualities | ○ | ○ | ○ | ○ | ○ |
| Your own supervision expertise | ○ | ○ | ○ | ○ | ○ |
| Training results | ○ | ○ | ○ | ○ | ○ |
| The strength of the sector's efforts to involve experts in enforcement | ○ | ○ | ○ | ○ | ○ |

12. Please evaluate the strength of the penalties imposed by the regulatory authorities at this level on the coal mining enterprises in their jurisdictions, considering the local situation

|  | Very little intensity | Low intensity | Average intensity | Powerful intensity | Great intensity |
| --- | --- | --- | --- | --- | --- |
| Total intensity of penalties | ○ | ○ | ○ | ○ | ○ |
| Intensity of administrative fines | ○ | ○ | ○ | ○ | ○ |
| Intensity of being ordered to stop production for rectification | ○ | ○ | ○ | ○ | ○ |
| Intensity of correspondence and interviews | ○ | ○ | ○ | ○ | ○ |

13. Please evaluate the effectiveness of regulation of coal mines in your jurisdiction at all levels of supervision

|  | Poor results | Less effective | Average results | Good results | Works very well |
| --- | --- | --- | --- | --- | --- |
| Overall Regulatory Outcomes | ○ | ○ | ○ | ○ | ○ |
| Identifying and solving problems | ○ | ○ | ○ | ○ | ○ |
| Examine and eliminate hidden dangers | ○ | ○ | ○ | ○ | ○ |
| Conveying safety awareness | ○ | ○ | ○ | ○ | ○ |

14. Do you think that the existing supervision equipment and supervision means can meet the needs of production safety supervision？

| ○Does not meet any needs ○Can meet 25% of demand ○Can meet 50% of needs ○Can meet 75% of needs ○Can meet 100% of needs |
| --- |

15. Please evaluate the quality of the risk classification and control of coal mines in your jurisdiction

|  | Very poor | poor | Average | Good | Very good |
| --- | --- | --- | --- | --- | --- |
| Overall work quality of risk classification and management | ○ | ○ | ○ | ○ | ○ |
| Work quality of risk identification and assessment | ○ | ○ | ○ | ○ | ○ |
| Work quality of risk management measures | ○ | ○ | ○ | ○ | ○ |
| Work quality of risk control safeguards (staffing, financial support, emphasis) | ○ | ○ | ○ | ○ | ○ |

16. Please evaluate the quality of the work on the investigation and management of hidden dangers in coal mines in your jurisdiction

|  | Very poor | poor | Average | Good | Very good |
| --- | --- | --- | --- | --- | --- |
| The overall work quality of hazard identification and management | ○ | ○ | ○ | ○ | ○ |
| Work quality of hidden dangers identification | ○ | ○ | ○ | ○ | ○ |
| Work quality of hidden hazards management | ○ | ○ | ○ | ○ | ○ |
| Work quality of supervisory and management on hidden danger rectification | ○ | ○ | ○ | ○ | ○ |

17. Please evaluate the development and implementation of regulations for coal mines in your jurisdiction

|  | Very bad | Not good | Average | Good | Very good |
| --- | --- | --- | --- | --- | --- |
| The overall quality of the corporate regulations | ○ | ○ | ○ | ○ | ○ |
| Integrity of corporate regulations | ○ | ○ | ○ | ○ | ○ |
| Operability of corporate rules and regulations | ○ | ○ | ○ | ○ | ○ |

18. Please evaluate the quality of the following work in the coal mines in your jurisdiction

|  | Very poor | poor | Average | Good | Very good |
| --- | --- | --- | --- | --- | --- |
| Overall quality of corporate security management | ○ | ○ | ○ | ○ | ○ |
| The quality of technical plan developed by technicians | ○ | ○ | ○ | ○ | ○ |
| Quality of site management by managers | ○ | ○ | ○ | ○ | ○ |
| Quality of work on site by frontline staff in the underground | ○ | ○ | ○ | ○ | ○ |

19. Based on your understanding, when rectifying problems in the coal mines under your jurisdiction, can you "learn from the past" to avoid the recurrence of the same type of problems?

| ○Did a very bad job ○Not well done ○Generally well done ○Doing quite well ○Well done |
| --- |

20. To the best of your knowledge, please rate the quality of the following aspects of the work of the coal mines in your jurisdiction

|  | Very poor | poor | Average | Good | Very good |
| --- | --- | --- | --- | --- | --- |
| The quality of what companies identify general problems and hazards in a timely manner | ○ | ○ | ○ | ○ | ○ |
| The quality of what companies identify major problems and hazards in a timely manner | ○ | ○ | ○ | ○ | ○ |
| The quality of what companies could timely work out effective control plans and corrective measures for potential hazards | ○ | ○ | ○ | ○ | ○ |
| The quality of what all levels of the enterprise can implement the work requirements of the program measures in a timely manner | ○ | ○ | ○ | ○ | ○ |
| The quality of what companies timely feedback all levels of the business on problems in the rectification process | ○ | ○ | ○ | ○ | ○ |

21. To the best of your knowledge, please rate the following in relation to coal mines in your jurisdiction.

|  | Definitely not | None | Hardly | Sometimes there are | Frequently |
| --- | --- | --- | --- | --- | --- |
| Does the company have an urge to overstaff the well | ○ | ○ | ○ | ○ | ○ |
| Does the company have an urge to conceal the location of the operation | ○ | ○ | ○ | ○ | ○ |

22. Please evaluate the coal mine managers in your jurisdiction in the light of your knowledge

|  | Very poor | poor | Average | Good | Very good |
| --- | --- | --- | --- | --- | --- |
| How well do they manage safety? | ○ | ○ | ○ | ○ | ○ |
| How is their corporate leadership? | ○ | ○ | ○ | ○ | ○ |
| How are their basic qualities? | ○ | ○ | ○ | ○ | ○ |

23. How well do you consider yourself to be able to identify major problems and hazards in the regulatory process?

| ○Very poor | ○Poor | ○Average | ○Good | ○Very good |
| --- | --- | --- | --- | --- |

24. In the course of daily supervision, are there any major hazards found and strict penalties not imposed on the enterprises for other reasons?

| ○Frequent | o will appear | ○Special cases may occur | ○Does not appear | o Absolutely not |
| --- | --- | --- | --- | --- |

Appendix B

Table. B-1 Expert scoring data on the importance of subsystems, level 1 indicators

|  | | Specialist A  （l,g,h） | Specialist B  （l,g,h） | Specialist C  （l,g,h） | Specialist D  （l,g,h） | Specialist E  （l,g,h） |
| --- | --- | --- | --- | --- | --- | --- |
| A Enterprise management level | | 50,55,60 | 50,65,80 | 50,60,70 | 40,45,50 | 40,45,50 |
| B Government Regulatory Level | | 30,35,40 | 5,14,20 | 15,20,30 | 40,45,50 | 30,35,40 |
| C Front-line employee level | | 15,17,20 | 15,23,30 | 30,40,50 | 10,15,20 | 20,23,30 |
| E External factor Level | | 20,25,30 | 10,20,30 | 40,42,45 | 20,25,30 | 10,11,12 |
| A Enterprise management level | AA | 50,55,60 | 40,45,50 | 70,75,80 | 60,65,70 | 40,45,50 |
|  | AB | 20,25,30 | 10,15,20 | 10,15,20 | 15,17,20 | 30,35,40 |
|  | AC | 30,33,35 | 50,60,70 | 30,40,50 | 30,35,40 | 30,35,40 |
| B Government Regulatory Level | BA | 50,53,55 | 30,35,40 | 40,45,50 | 60,65,70 | 40,45,50 |
|  | BB | 30,33,35 | 50,65,80 | 40,50,60 | 15,25,30 | 30,33,35 |
|  | BC | 20,23,25 | 20,25,30 | 30,32,35 | 15,24,30 | 20,25,30 |
| C Front-line employee level | CA | 20,25,30 | 50,65,80 | 15,17,20 | 20,25,30 | 50,55,60 |
|  | CB | 60,65,70 | 30,35,40 | 35,37,40 | 60,65,70 | 40,45,50 |
|  | CC | 15,17,20 | 20,25,30 | 30,35,40 | 20,25,30 | 10,13,15 |
| E External factor Level | EA | 30,33,35 | 40,50,60 | 40,50,60 | 25,30,35 | 30,35,40 |
|  | EB | 40,43,45 | 20,25,30 | 30,40,50 | 40,45,50 | 40,45,50 |
|  | EC | 10,12,15 | 30,35,40 | 10,13,15 | 15,17,20 | 30,35,40 |
|  | ED | 20,23,25 | 10,15,20 | 15,17,20 | 15,17,20 | 10,13,15 |

Appendix C

Table. C-1 Weight calculation results of subsystem w_1_and first-level index w_2_

| **The subsystem** | **First-level index** | **I（left）** | **I（right）** | **I** | **Weight** | **Weight type** |
| --- | --- | --- | --- | --- | --- | --- |
| A Enterprise management level |  | 0.3507 | 0.4916 | 0.4211 | 49.96% | E_1_ |
| B Government Regulatory Level |  | 0.1891 | 0.2795 | 0.2343 | 27.80% | E_1_ |
| C Front-line employee level |  | 0.1466 | 0.2283 | 0.1874 | 22.24% | E_1_ |
| E External factor Level |  | 0.1567 | 0.2293 | 0.1930 | 22.90% | The additional weight，E1 |
| A Enterprise management level | AA Safety management quality | 0.4307 | 0.5453 | 0.4880 | 47.87% | w_2_ |
|  | AB Management personnel operating pressure | 0.1524 | 0.2161 | 0.1843 | 18.08% | w_2_ |
|  | AC safety input | 0.2956 | 0.3987 | 0.3472 | 34.05% | w_2_ |
| B Government Regulatory Level | BA Regulatory Achievements | 0.3781 | 0.4806 | 0.4294 | 42.05% | w_2_ |
|  | BB Safety regulatory economic aspects | 0.3042 | 0.4231 | 0.3636 | 35.61% | w_2_ |
|  | BC Regulatory pressure | 0.1917 | 0.2647 | 0.2282 | 22.35% | w_2_ |
| C Front-line employee level | CA Safety atmosphere | 0.2943 | 0.4019 | 0.3481 | 34.16% | w_2_ |
|  | CB The knowledge and skills that should know and should master | 0.4050 | 0.5092 | 0.4571 | 44.86% | w_2_ |
|  | CC Worker safety status | 0.1807 | 0.2468 | 0.2138 | 20.98% | w_2_ |
| E External factor Level | EA Ore prices | 0.2874 | 0.3969 | 0.3422 | 33.38% | w_2_ |
|  | EB Industry policies adjustment | 0.2910 | 0.3919 | 0.3415 | 33.32% | w_2_ |
|  | EC Production safety accident occur with great impact | 0.1638 | 0.2244 | 0.1941 | 18.94% | w_2_ |
|  | ED Underground geological conditions ofenterprises have drastic changes | 0.1228 | 0.1717 | 0.1472 | 14.36% | w_2_ |

Table. C-2 Weight calculation results of Secondary index w_3_

| **Weight** | | | | | | | |
| --- | --- | --- | --- | --- | --- | --- | --- |
|  | **Weight of 1**  **（CRITIC method）** | **Weight of 2**  **（Entropy weight method）** | **Weight of 3**  **（Rough set -Risk）** | **Weight of 4**  **（Rough set -Neg）** | **Weight of 5**  **（Rough set -Pos）** | **The comprehensive weights** | **Weight type** |
| AA | | | | | | | |
| **Weight method ratio** | 19.59% | 46.44% | 2.95% | 10.17% | 20.86% |  |  |
| AA1 Work quality of risk grading control | 10.74% | 10.13% | 12.77% | 12.81% | 13.07% | 11.21% | w_3_ |
| AA2 Work quality of hidden peril identification and governance | 12.29% | 11.52% | 13.17% | 11.80% | 14.78% | 12.43% | w_3_ |
| AA3 General quality of regulations | 10.43% | 9.65% | 12.52% | 12.98% | 12.20% | 10.76% | w_3_ |
| AA4 Management ability of management personnel | 13.45% | 13.75% | 12.41% | 13.16% | 11.71% | 13.17% | w_3_ |
| AA5 Reward and Punishment Mechanism | 14.59% | 17.72% | 11.90% | 10.87% | 10.50% | 14.73% | w_3_ |
| AA6 Quality of safety training | 12.94% | 9.28% | 12.57% | 11.48% | 15.40% | 11.60% | w_3_ |
| AA7 Quality of site management | 11.08% | 14.24% | 12.11% | 14.10% | 11.09% | 12.89% | w_3_ |
| AA8 Improvement efforts of feedack | 14.48% | 13.70% | 12.54% | 12.81% | 11.24% | 13.22% | w_3_ |
| AB | | | | | | | |
| **Weight method ratio** | 11.56% | 41.58% | 12.96% | 22.53% | 11.37% |  |  |
| AB1 Corporate profitability pressure of management personnel | 27.64% | 25.19% | 31.94% | 24.76% | 27.09% | 26.46% | w_3_ |
| AB2 Corporate stability pressure of management personnel | 38.35% | 55.42% | 35.35% | 36.33% | 41.42% | 44.95% | w_3_ |
| AB3 Safety pressure of management personnel | 34.01% | 19.40% | 32.71% | 38.91% | 31.49% | 28.58% | w_3_ |
| AC | | | | | | | |
| **Weight method ratio** | 7.23% | 41.61% | 1.51% | 23.26% | 26.39% |  |  |
| AC1 Daily safety engineering investment | 40.78% | 17.53% | 31.08% | 34.09% | 34.86% | 27.84% | w_3_ |
| AC2 Enterprise output value | 30.12% | 38.48% | 35.38% | 33.63% | 33.46% | 35.38% | w_3_ |
| AC3 Enterprise surplus | 29.10% | 43.98% | 33.53% | 32.28% | 31.68% | 36.78% | w_3_ |
| BA | | | | | | | |
| **Weight method ratio** | 8.60% | 76.46% | 1.49% | 3.41% | 10.04% |  |  |
| BA1 Frequency of supervision | 15.23% | 6.97% | 11.96% | 10.91% | 10.03% | 8.20% | w_3_ |
| BA2 Punishment mechanism | 13.18% | 13.65% | 11.11% | 11.55% | 12.98% | 13.43% | w_3_ |
| BA3 Supervision capability | 14.17% | 26.11% | 13.08% | 11.88% | 11.91% | 22.98% | w_3_ |
| BA4 Supervision initiative | 12.90% | 12.67% | 11.63% | 13.71% | 9.48% | 12.39% | w_3_ |
| BA5 Modern Supervision methods | 15.47% | 18.43% | 12.97% | 11.55% | 12.26% | 17.24% | w_3_ |
| BA6 Government supervise and identify hidden peril | 9.58% | 7.07% | 12.68% | 12.58% | 14.14% | 8.27% | w_3_ |
| BA7 Governments supervise and find problems | 8.94% | 6.38% | 13.64% | 14.11% | 15.85% | 7.92% | w_3_ |
| BA8 Safety awareness promotion | 10.52% | 8.72% | 12.92% | 13.71% | 13.35% | 9.57% | w_3_ |
| BC | | | | | | | |
| **Weight method ratio** | 8.71% | 82.80% | 2.40% | 1.23% | 4.87% |  |  |
| BC1 Social stability pressure | 57.62% | 85.14% | 46.01% | 47.15% | 44.31% | 79.35% | w_3_ |
| BC2 Accident accountability pressure | 42.38% | 14.86% | 53.99% | 52.85% | 55.69% | 20.65% | w_3_ |
| CA | | | | | | | |
| **Weight method ratio** | 17.95% | 47.71% | 14.54% | 13.78% | 6.03% |  |  |
| CA1 Stop unsafe behavior of workmates | 17.60% | 9.31% | 16.02% | 16.61% | 14.15% | 13.07% | w_3_ |
| CA2 Report safety risks of other positions | 14.73% | 6.74% | 16.22% | 16.88% | 19.53% | 11.72% | w_3_ |
| CA3 Reveres all kinds of safety regulations | 21.75% | 12.23% | 16.80% | 16.61% | 15.33% | 15.39% | w_3_ |
| CA4 Income satisfaction | 17.27% | 26.44% | 17.15% | 17.72% | 16.89% | 21.66% | w_3_ |
| CA5 Working environment Satisfaction | 14.76% | 23.68% | 17.47% | 15.57% | 20.17% | 19.85% | w_3_ |
| CA6 Work intensity satisfaction | 13.89% | 21.60% | 16.34% | 16.61% | 13.92% | 18.30% | w_3_ |
| CB | | | | | | | |
| **Weight method ratio** | 24.14% | 25.04% | 20.96% | 6.21% | 23.65% |  |  |
| CB1 Detect safety risks | 48.70% | 49.89% | 52.66% | 45.18% | 48.39% | 49.53% | w_3_ |
| CB2 Self-management ability | 51.30% | 50.11% | 47.34% | 54.82% | 51.61% | 50.47% | w_3_ |
| CC | | | | | | | |
| **Weight method ratio** | 16.38% | 31.78% | 2.09% | 39.39% | 10.36% |  |  |
| CC1 Safety concept | 36.03% | 34.39% | 36.29% | 33.51% | 33.87% | 34.30% | w_3_ |
| CC2 Worker safety awareness | 32.35% | 33.73% | 30.86% | 33.51% | 32.80% | 33.26% | w_3_ |
| CC3 Workers "three violations" | 31.62% | 31.88% | 32.86% | 32.98% | 33.33% | 32.44% | w_3_ |

Table. C-3 Weight calculation results of AC BA tertiary index w_4_

| Weight | | | | | | | |
| --- | --- | --- | --- | --- | --- | --- | --- |
|  | **Weight of 1**  **（CRITIC method）** | **Weight of 2**  **（Entropy weight method）** | **Weight of 3**  **（Rough set -Risk）** | **Weight of 4**  **（Rough set -Neg）** | **Weight of 5**  **（Rough set -Pos）** | **The comprehensive weights** | **Weight type** |
| AC1 | | | | | | | |
| **Weight method ratio** | 2.89% | 45.96% | 44.84% | 3.15% | 3.15% |  |  |
| AC11 Investment of few technologies and equipment | 53.27% | 64.84% | 30.10% | 46.58% | 46.58% | 47.78% | w_4_ |
| AC12 Investment on safety training input | 46.73% | 35.16% | 69.90% | 53.42% | 53.42% | 52.22% | w_4_ |
| AC2 | | | | | | | |
| **Weight method ratio** | 9.60% | 76.35% | 3.00% | 1.29% | 9.76% |  |  |
| AC21 Insufficiency rate of operating personnel | 25.08% | 31.21% | 25.10% | 25.25% | 23.96% | 29.66% | w_4_ |
| AC22 Unsaturation rate of enterprise operating site | 24.40% | 28.14% | 27.06% | 23.90% | 27.10% | 27.60% | w_4_ |
| AC23 The impulse of concealing the operating site of the enterprise | 22.12% | 8.98% | 23.11% | 25.25% | 21.47% | 12.09% | w_4_ |
| AC24 The impulse of overmanned operation in the well of the enterprise | 28.40% | 31.67% | 24.73% | 25.60% | 27.47% | 30.66% | w_4_ |
| AC3 | | | | | | | |
| **Weight method ratio** | 25.86% | 17.57% | 43.92% | 1.89% | 10.76% |  |  |
| AC31 Employee salary | 42.81% | 37.07% | 33.13% | 33.94% | 34.10% | 36.44% | w_4_ |
| AC32 Superior financial assistance | 29.94% | 33.69% | 34.29% | 32.13% | 32.73% | 32.85% | w_4_ |
| AC33 Superior financial pressure | 27.25% | 29.25% | 32.58% | 33.94% | 33.18% | 30.71% | w_4_ |
| BA1 | | | | | | | |
| **Weight method ratio** | 0.80% | 30.65% | 24.48% | 37.37% | 6.70% |  |  |
| BA12 Frequency of Provincial supervision | 32.84% | 42.14% | 36.82% | 28.75% | 39.86% | 35.60% | w_4_ |
| BA13 Frequency of municipal supervision | 20.66% | 17.14% | 27.98% | 41.68% | 27.50% | 29.68% | w_4_ |
| BA14 Frequency of County-level supervision | 46.51% | 40.72% | 35.20% | 29.58% | 32.64% | 34.71% | w_4_ |
| BA2 | | | | | | | |
| **Weight method ratio** | 23.08% | 60.01% | 3.51% | 0.98% | 12.42% |  |  |
| BA21 Administrative penalty | 21.34% | 37.12% | 28.28% | 26.42% | 30.06% | 32.18% | w_4_ |
| BA22 Production suspension orders | 17.76% | 17.21% | 25.50% | 22.90% | 27.59% | 18.97% | w_4_ |
| BA23 Circularization and appointment talks | 23.28% | 19.88% | 27.50% | 24.25% | 29.22% | 22.13% | w_4_ |
| BA24 Penalties for major hidden peril | 37.62% | 25.80% | 18.72% | 26.42% | 13.13% | 26.71% | w_4_ |
| BA3 | | | | | | | |
| **Weight method ratio** | 46.53% | 34.98% | 2.37% | 13.81% | 2.31% |  |  |
| BA31 Experts' participation | 14.34% | 5.66% | 15.07% | 16.44% | 12.48% | 11.57% | w_4_ |
| BA32 Competency of supervision cadres | 20.41% | 30.24% | 23.84% | 23.84% | 27.77% | 24.57% | w_4_ |
| BA33 Professional skills of supervision cadres | 16.81% | 28.52% | 23.60% | 24.53% | 28.58% | 22.41% | w_4_ |
| BA34 Supervision business training | 17.74% | 23.02% | 20.19% | 20.09% | 19.71% | 20.02% | w_4_ |
| BA35 Capacities to detect significant hazards | 30.70% | 12.56% | 17.30% | 15.09% | 11.45% | 21.44% | w_4_ |

Table.C-4 Weight calculation results of AA tertiary index w_41_ by questionnaire Ⅱ survey data

| **Weight** | | | | | | | |
| --- | --- | --- | --- | --- | --- | --- | --- |
|  | **Weight of 1**  **（CRITIC method）** | **Weight of 2**  **（Entropy weight method）** | **Weight of 3**  **（Rough set -Risk）** | **Weight of 4**  **（Rough set -Neg）** | **Weight of 5**  **（Rough set -Pos）** | **The comprehensive weights** | **Weight type** |
| AA1 | | | | | | | |
| **Weight method ratio** | 24.59% | 21.05% | 18.33% | 18.65% | 17.39% |  |  |
| AA11 Safety risk identification and assessment | 29.15% | 31.56% | 35.87% | 33.33% | 35.55% | 32.78% | Questionnaire Ⅱ, w_41_ |
| AA12 Safety risk control measures | 27.28% | 24.93% | 37.79% | 32.88% | 37.04% | 31.45% | Questionnaire Ⅱ, w_41_ |
| AA13 Risk control safeguards | 43.56% | 43.51% | 26.34% | 33.79% | 27.40% | 35.76% | Questionnaire Ⅱ, w_41_ |
| AA2 | | | | | | | |
| **Weight method ratio** | 54.69% | 24.21% | 10.82% | 2.92% | 7.35% |  |  |
| AA21 Work quality of hidden peril identification | 37.28% | 30.35% | 33.71% | 34.41% | 31.68% | 34.72% | Questionnaire Ⅱ, w_41_ |
| AA22 Work quality of hidden peril governance | 29.94% | 35.72% | 33.50% | 33.02% | 34.39% | 32.15% | Questionnaire Ⅱ, w_41_ |
| AA23 Work quality of hidden peril rectification supervision and management | 32.78% | 33.93% | 32.79% | 32.57% | 33.93% | 33.14% | Questionnaire Ⅱ, w_41_ |
| AA3 | | | | | | | |
| **Weight method ratio** | 5.41% | 24.94% | 5.17% | 9.32% | 55.17% |  |  |
| AA31 Completeness of regulations and policies within the enterprise | 51.85% | 53.74% | 51.81% | 47.60% | 55.12% | 53.73% | Questionnaire Ⅱ, w_41_ |
| AA32 Operability of the regulations and policies of the enterprise | 48.15% | 46.26% | 48.19% | 52.40% | 44.88% | 46.27% | Questionnaire Ⅱ, w_41_ |
| AA4 | | | | | | | |
| **Weight method ratio** | 11.01% | 15.52% | 0.46% | 2.29% | 70.72% |  |  |
| AA41 Competency of management personnel | 46.38% | 45.78% | 50.77% | 51.71% | 57.48% | 54.28% | Questionnaire Ⅱ, w_41_ |
| AA42 Management leadership | 53.62% | 54.22% | 49.23% | 48.29% | 42.52% | 45.72% | Questionnaire Ⅱ, w_41_ |
| AA5 | | | | | | | |
| **Weight method ratio** | 28.29% | 8.58% | 15.34% | 15.78% | 32.02% |  |  |
| AA51 Implementation of regulations and policies | 29.69% | 31.63% | 33.73% | 33.64% | 35.33% | 32.90% | Questionnaire Ⅱ, w_41_ |
| AA52 Punishments Mechanism | 31.36% | 20.72% | 30.72% | 32.73% | 25.78% | 28.78% | Questionnaire Ⅱ, w_41_ |
| AA53 Reward Mechanism | 38.95% | 47.65% | 35.55% | 33.64% | 38.89% | 38.32% | Questionnaire Ⅱ, w_41_ |
| AA6 | | | | | | | |
| **Weight method ratio** | 1.05% | 35.77% | 18.30% | 38.81% | 6.07% |  |  |
| AA61 Trainer resources | 30.66% | 35.67% | 29.17% | 31.34% | 35.05% | 32.71% | Questionnaire Ⅱ, w_41_ |
| AA62 Attractiveness of training method | 30.12% | 34.83% | 33.73% | 36.44% | 28.93% | 34.84% | Questionnaire Ⅱ, w_41_ |
| AA63 Training assessment strength | 39.23% | 29.51% | 37.11% | 32.21% | 36.02% | 32.45% | Questionnaire Ⅱ, w_41_ |
| AA7 | | | | | | | |
| **Weight method ratio** | 23.22% | 23.20% | 7.28% | 23.15% | 23.15% |  |  |
| AA71 Technical solution quality | 54.58% | 53.97% | 68.37% | 52.40% | 52.40% | 54.43% | Questionnaire Ⅱ, w_41_ |
| AA72 Site construction quality | 45.42% | 46.03% | 31.63% | 47.60% | 47.60% | 45.57% | Questionnaire Ⅱ, w_41_ |
| AA8 | | | | | | | |
| **Weight method ratio** | 15.96% | 40.39% | 3.62% | 3.38% | 36.64% |  |  |
| AA81 In-time identification of common problems | 19.84% | 18.05% | 19.78% | 20.83% | 13.38% | 16.78% | Questionnaire Ⅱ, w_41_ |
| AA82 In-time identification of major problems | 20.90% | 19.41% | 20.06% | 19.45% | 22.83% | 20.93% | Questionnaire Ⅱ, w_41_ |
| AA83 In-time effective solution | 16.58% | 16.06% | 19.46% | 19.72% | 15.55% | 16.21% | Questionnaire Ⅱ, w_41_ |
| AA84 In-time implementation of the solution | 21.28% | 22.31% | 20.25% | 19.99% | 24.78% | 22.90% | Questionnaire Ⅱ, w_41_ |
| AA85 In-time follow up of the rectification | 21.40% | 24.17% | 20.45% | 19.99% | 23.46% | 23.19% | Questionnaire Ⅱ, w_41_ |

Table. C-5 Weight calculation results of AA tertiary index w_42_ by questionnaireⅠand Ⅲ survey data

| **Weight** | | | | | | | | |
| --- | --- | --- | --- | --- | --- | --- | --- | --- |
|  | **Weight of 1**  **（CRITIC method）** | **Weight of 2**  **（Entropy weight method）** | **Weight of 3**  **（Rough set -Risk）** | **Weight of 4**  **（Rough set -Neg）** | **Weight of 5**  **（Rough set -Pos）** | **The comprehensive weights** | **Weight type** | |
| AA1 | | | | | | | | |
| **Weight method ratio** | 8.00% | 12.79% | 22.02% | 55.93% | 1.26% |  | |  |
| AA11 Safety risk identification and assessment | 34.79% | 42.60% | 38.95% | 34.27% | 34.88% | 36.42% | | Questionnaire Ⅰ, w_42_ |
| AA12 Safety risk control measures | 32.06% | 36.74% | 38.99% | 34.27% | 34.88% | 35.46% | | Questionnaire Ⅰ, w_42_ |
| AA13 Risk control safeguards | 33.15% | 20.66% | 22.06% | 31.46% | 30.24% | 28.13% | | Questionnaire Ⅰ, w_42_ |
| AA2 | | | | | | | | |
| **Weight method ratio** | 33.83% | 0.84% | 27.25% | 0.43% | 37.66% |  | |  |
| AA21 Work quality of hidden peril identification | 28.86% | 34.80% | 33.66% | 29.32% | 39.18% | 34.11% | | Questionnaire Ⅰ, w_42_ |
| AA22 Work quality of hidden peril governance | 34.26% | 32.61% | 34.57% | 33.83% | 33.01% | 33.86% | | Questionnaire Ⅰ, w_42_ |
| AA23 Work quality of hidden peril rectification supervision and management | 36.88% | 32.60% | 31.77% | 36.85% | 27.81% | 32.04% | | Questionnaire Ⅰ, w_42_ |
| AA3 | | | | | | | | |
| **Weight method ratio** | 20.82% | 23.98% | 17.70% | 18.74% | 18.74% |  | |  |
| AA31 Completeness of regulations and policies within the enterprise | 51.18% | 50.56% | 48.47% | 51.43% | 162.53% | 50.11% | | Questionnaire Ⅰ, w_42_ |
| AA32 Operability of the regulations and policies of the enterprise | 48.82% | 49.44% | 51.53% | 48.57% | 172.09% | 49.89% | | Questionnaire Ⅰ, w_42_ |
| AA4 | | | | | | | | |
| **Weight method ratio** | 23.90% | 22.36% | 5.40% | 24.24% | 24.10% |  | |  |
| AA41 Competency of management personnel | 47.80% | 45.08% | 60.70% | 50.00% | 51.43% | 49.30% | | Questionnaire Ⅰ, w_42_ |
| AA42 Management leadership | 52.20% | 54.92% | 39.30% | 50.00% | 48.57% | 50.70% | | Questionnaire Ⅰ, w_42_ |
| AA5 | | | | | | | | |
| **Weight method ratio** | 20.59% | 4.22% | 16.78% | 17.12% | 41.29% |  | |  |
| AA51 Implementation of regulations and policies | 31.73% | 34.84% | 50.00% | 44.99% | 53.20% | 46.06% | |  |
| AA52 Punishments Mechanism | 33.13% | 26.38% | 25.42% | 27.73% | 22.27% | 26.14% | | Questionnaire Ⅲ, w_42_ |
| AA53 Reward Mechanism | 35.14% | 38.78% | 24.58% | 27.29% | 24.53% | 27.80% | | Questionnaire Ⅲ, w_42_ |
| AA6 | | | | | | | | |
| **Weight method ratio** | 12.78% | 11.17% | 1.22% | 58.06% | 16.77% |  | |  |
| AA61 Trainer resources | 30.34% | 24.75% | 31.84% | 31.91% | 34.31% | 31.31% | | Questionnaire Ⅲ, w_42_ |
| AA62 Attractiveness of training method | 31.72% | 41.61% | 34.98% | 33.49% | 36.01% | 34.62% | | Questionnaire Ⅲ, w_42_ |
| AA63 Training assessment strength | 37.93% | 33.65% | 33.18% | 34.59% | 29.68% | 34.07% | | Questionnaire Ⅲ, w_42_ |
| AA7 | | | | | | | | |
| **Weight method ratio** | 21.83% | 23.96% | 31.91% | 4.13% | 18.16% |  | |  |
| AA71 Technical solution quality | 54.63% | 54.82% | 44.58% | 52.14% | 45.72% | 49.75% | | Questionnaire Ⅰ, w_42_ |
| AA72 Site construction quality | 45.37% | 45.18% | 55.42% | 47.86% | 54.28% | 50.25% | | Questionnaire Ⅰ, w_42_ |
| AA8 | | | | | | | | |
| **Weight method ratio** | 2.84% | 24.19% | 39.87% | 21.25% | 11.85% |  | |  |
| AA81 In-time identification of common problems | 20.26% | 16.56% | 18.54% | 18.65% | 17.23% | 17.98% | | Questionnaire Ⅰ, w_42_ |
| AA82 In-time identification of major problems | 32.55% | 36.92% | 20.73% | 19.75% | 22.93% | 25.03% | | Questionnaire Ⅰ, w_42_ |
| AA83 In-time effective solution | 16.39% | 15.66% | 20.80% | 19.75% | 19.32% | 19.03% | | Questionnaire Ⅰ, w_42_ |
| AA84 In-time implementation of the solution | 13.15% | 12.33% | 19.04% | 21.52% | 18.24% | 17.68% | | Questionnaire Ⅰ, w_42_ |
| AA85 In-time follow up of the rectification | 17.65% | 18.53% | 20.88% | 20.32% | 22.28% | 20.27% | | Questionnaire Ⅰ, w_42_ |

Table. C-6 Weight calculation results of AA tertiary index w_4_

| **Index** | **Process Weight 1** | **Note 1** | **The weight of process weight 1** | **Process Weight 2** | **Note 2** | **The weight of process weight2** | **The comprehensive weights** | **Weight type** |
| --- | --- | --- | --- | --- | --- | --- | --- | --- |
| AA1 | | | | | | | |  |
| AA11 Safety risk identification and assessment | 32.78% | Questionnaire Ⅱ, w_41_ | 0.4 | 36.42% | Questionnaire Ⅰ, w_42_ | 0.6 | 34.96% | w_4_ |
| AA12 Safety risk control measures | 31.45% | Questionnaire Ⅱ, w_41_ | 0.4 | 35.46% | Questionnaire Ⅰ, w_42_ | 0.6 | 33.86% | w_4_ |
| AA13 Risk control safeguards | 35.76% | Questionnaire Ⅱ, w_41_ | 0.4 | 28.13% | Questionnaire Ⅰ, w_42_ | 0.6 | 31.18% | w_4_ |
| AA2 | | | | | | | |  |
| AA21 Work quality of hidden peril identification | 34.72% | Questionnaire Ⅱ, w_41_ | 0.4 | 34.11% | Questionnaire Ⅰ, w_42_ | 0.6 | 34.35% | w_4_ |
| AA22 Work quality of hidden peril governance | 32.15% | Questionnaire Ⅱ, w_41_ | 0.4 | 33.86% | Questionnaire Ⅰ, w_42_ | 0.6 | 33.18% | w_4_ |
| AA23 Work quality of hidden peril rectification supervision and management | 33.14% | Questionnaire Ⅱ, w_41_ | 0.4 | 32.04% | Questionnaire Ⅰ, w_42_ | 0.6 | 32.48% | w_4_ |
| AA3 | | | | | | | |  |
| AA31 Completeness of regulations and policies within the enterprise | 53.73% | Questionnaire Ⅱ, w_41_ | 0.4 | 50.11% | Questionnaire Ⅰ, w_42_ | 0.6 | 51.56% | w_4_ |
| AA32 Operability of the regulations and policies of the enterprise | 46.27% | Questionnaire Ⅱ, w_41_ | 0.4 | 49.89% | Questionnaire Ⅰ, w_42_ | 0.6 | 48.44% | w_4_ |
| AA4 | | | | | | | |  |
| AA41 Competency of management personnel | 54.28% | Questionnaire Ⅱ, w_41_ | 0.4 | 49.30% | Questionnaire Ⅰ, w_42_ | 0.6 | 51.29% | w_4_ |
| AA42 Management leadership | 45.72% | Questionnaire Ⅱ, w_41_ | 0.4 | 50.70% | Questionnaire Ⅰ, w_42_ | 0.6 | 48.71% | w_4_ |
| AA5 | | | | | | | |  |
| AA51 Implementation of regulations and policies | 32.90% | Questionnaire Ⅱ, w_41_ | 0.45 | 46.06% | Questionnaire Ⅲ, w_42_ | 0.55 | 40.14% | w_4_ |
| AA52 Punishments Mechanism | 28.78% | Questionnaire Ⅱ, w_41_ | 0.45 | 26.14% | Questionnaire Ⅲ, w_42_ | 0.55 | 27.33% | w_4_ |
| AA53 Reward Mechanism | 38.32% | Questionnaire Ⅱ, w_41_ | 0.45 | 27.80% | Questionnaire Ⅲ, w_42_ | 0.55 | 32.53% | w_4_ |
| AA6 | | | | | | | |  |
| AA61 Trainer resources | 32.71% | Questionnaire Ⅱ, w_41_ | 0.45 | 31.31% | Questionnaire Ⅲ, w_42_ | 0.55 | 31.94% | w_4_ |
| AA62 Attractiveness of training method | 34.84% | Questionnaire Ⅱ, w_41_ | 0.45 | 34.62% | Questionnaire Ⅲ, w_42_ | 0.55 | 34.72% | w_4_ |
| AA63 Training assessment strength | 32.45% | Questionnaire Ⅱ, w_41_ | 0.45 | 34.07% | Questionnaire Ⅲ, w_42_ | 0.55 | 33.34% | w_4_ |
| AA7 | | | | | | | |  |
| AA71 Technical solution quality | 54.43% | Questionnaire Ⅱ, w_41_ | 0.4 | 49.75% | Questionnaire Ⅰ, w_42_ | 0.6 | 51.62% | w_4_ |
| AA72 Site construction quality | 45.57% | Questionnaire Ⅱ, w_41_ | 0.4 | 50.25% | Questionnaire Ⅰ, w_42_ | 0.6 | 48.38% | w_4_ |
| AA8 | | | | | | | |  |
| AA81 In-time identification of common problems | 16.78% | Questionnaire Ⅱ, w_41_ | 0.4 | 17.98% | Questionnaire Ⅰ, w_42_ | 0.6 | 17.50% | w_4_ |
| AA82 In-time identification of major problems | 20.93% | Questionnaire Ⅱ, w_41_ | 0.4 | 25.03% | Questionnaire Ⅰ, w_42_ | 0.6 | 23.39% | w_4_ |
| AA83 In-time effective solution | 16.21% | Questionnaire Ⅱ, w_41_ | 0.4 | 19.03% | Questionnaire Ⅰ, w_42_ | 0.6 | 17.90% | w_4_ |
| AA84 In-time implementation of the solution | 22.90% | Questionnaire Ⅱ, w_41_ | 0.4 | 17.68% | Questionnaire Ⅰ, w_42_ | 0.6 | 19.77% | w_4_ |
| AA85 In-time follow up of the rectification | 23.19% | Questionnaire Ⅱ, w_41_ | 0.4 | 20.27% | Questionnaire Ⅰ, w_42_ | 0.6 | 21.44% | w_4_ |

Table.C-6 Weight calculation results of index at each level E_i_

| The subsystem  （The weight of E_1_） | First-order influencing factor  （The weight of E_2_） | Secondary influencing factor | The weight of E_3_ | Tertiary influencing factor | The weight of E_4_ |
| --- | --- | --- | --- | --- | --- |
| A Enterprise management level（49.96%) | AA Safety management quality（23.92%） | AA1 | 2.6812% | AA11 | 0.9373% |
|  |  |  |  | AA12 | 0.9079% |
|  |  |  |  | AA13 | 0.8360% |
|  |  | AA2 | 2.9730% | AA21 | 1.0212% |
|  |  |  |  | AA22 | 0.9864% |
|  |  |  |  | AA23 | 0.9656% |
|  |  | AA3 | 2.5736% | AA31 | 1.3269% |
|  |  |  |  | AA32 | 1.2466% |
|  |  | AA4 | 3.1500% | AA41 | 1.6156% |
|  |  |  |  | AA42 | 1.5344% |
|  |  | AA5 | 3.5231% | AA51 | 1.4142% |
|  |  |  |  | AA52 | 0.9629% |
|  |  |  |  | AA53 | 1.1461% |
|  |  | AA6 | 2.7745% | AA61 | 0.8862% |
|  |  |  |  | AA62 | 0.9633% |
|  |  |  |  | AA63 | 0.9250% |
|  |  | AA7 | 3.0830% | AA71 | 1.5915% |
|  |  |  |  | AA72 | 1.4916% |
|  |  | AA8 | 3.1620% | AA81 | 0.5533% |
|  |  |  |  | AA82 | 0.7396% |
|  |  |  |  | AA83 | 0.5660% |
|  |  |  |  | AA84 | 0.6251% |
|  |  |  |  | AA85 | 0.6779% |
|  | AB Management personnel operating pressure（9.03%） | AB1 | 2.3903% |  |  |
|  |  | AB1 | 4.0606% |  |  |
|  |  | AB3 | 2.5818% |  |  |
|  | AC safety input（17.01%） | AC1 | 4.7364% | AC11 | 2.2630% |
|  |  |  |  | AC12 | 2.4733% |
|  |  | AC2 | 6.0192% | AC21 | 1.7853% |
|  |  |  |  | AC22 | 1.6613% |
|  |  |  |  | AC23 | 0.7277% |
|  |  |  |  | AC24 | 1.8455% |
|  |  | AC3 | 6.2573% | AC31 | 2.2802% |
|  |  |  |  | AC32 | 2.0555% |
|  |  |  |  | AC33 | 1.9216% |
| B Government Regulatory Level（27.8%） | BA Regulatory Achievements（11.69%） | BA1 | 0.9586% | BA12 | 0.3413% |
|  |  |  |  | BA13 | 0.2845% |
|  |  |  |  | BA14 | 0.3327% |
|  |  | BA2 | 1.5700% | BA21 | 0.5052% |
|  |  |  |  | BA22 | 0.2978% |
|  |  |  |  | BA23 | 0.3474% |
|  |  |  |  | BA24 | 0.4193% |
|  |  | BA3 | 2.6864% | BA31 | 0.3108% |
|  |  |  |  | BA32 | 0.6600% |
|  |  |  |  | BA33 | 0.6020% |
|  |  |  |  | BA34 | 0.5378% |
|  |  |  |  | BA35 | 0.5760% |
|  |  | BA4 | 1.4484% |  |  |
|  |  | BA5 | 2.0154% |  |  |
|  |  | BA6 | 0.9668% |  |  |
|  |  | BA7 | 0.9258% |  |  |
|  |  | BA8 | 1.1187% |  |  |
|  | BB Safety regulatory economic aspects  （9.90%） |  |  |  |  |
|  | BC Regulatory pressure  （6.21%） | BC1 | 4.9303% |  |  |
|  |  | BC2 | 1.2831% |  |  |
| C Front-line employee level（22.24%） | CA Safety atmosphere  （7.60%） | CA1 | 0.9927% |  |  |
|  |  | CA2 | 0.8902% |  |  |
|  |  | CA3 | 1.1690% |  |  |
|  |  | CA4 | 1.6452% |  |  |
|  |  | CA5 | 1.5077% |  |  |
|  |  | CA6 | 1.3900% |  |  |
|  | CB The knowledge and skills that should know and should master  （9.97%） | CB1 | 4.9405% |  |  |
|  |  | CB2 | 5.0343% |  |  |
|  | CC Worker safety status  （4.66%） | CC1 | 1.6001% |  |  |
|  |  | CC2 | 1.5516% |  |  |
|  |  | CC3 | 1.5133% |  |  |
| E External factor Level（22.9%） | EA Ore prices  （7.64%） |  |  |  |  |
|  | EB Industry policies adjustment  （7.63%） |  |  |  |  |
|  | EC Production safety accident occur with great impact  （4.34%） |  |  |  |  |
|  | ED Underground geological conditions ofenterprises have drastic changes  （3.29%） |  |  |  |  |
